# Cdk1 Deficiency Extends the Postnatal Window of Cardiomyocyte Proliferation and Restores Cardiac Function after Myocardial Infarction

**DOI:** 10.3390/ijms251910824

**Published:** 2024-10-09

**Authors:** Donya Mahiny, Ludger Hauck, Benny Premsingh, Daniela Grothe, Filio Billia

**Affiliations:** 1 Toronto General Hospital Research Institute, 100 College St., Toronto, ON M5G 1L7, Canada; donya.mahiny@uottawa.ca (D.M.); lpahauck@yahoo.com (L.H.); bennyprem.premsingh@uhn.ca (B.P.); daniela.grothe@uhn.ca (D.G.); 2 Division of Cardiology, University Health Network (UHN), 200 Elizabeth St., Toronto, ON M5G 2C4, Canada

**Keywords:** cardiac regeneration, cell cycle, heart failure, proliferation

## Abstract

Cyclin-dependent kinase 1 (*Cdk1*) is a master regulator of the G2-M transition between DNA replication and cell division. This study investigates the regulation of cardiomyocyte (CM) proliferation during the early neonatal period and following ischemic injury in adult mice. We analyzed cell cycle dynamics with the assessment of DNA synthesis, and cytokinesis in murine hearts during the first 15 days after birth. A distinct proliferative block was observed at 1 day, followed by a second wave of DNA synthesis at 4 days, leading to CM binucleation (CMBN) by day 5. Genome-wide mRNA profiling revealed the differential expression of cell cycle regulatory genes during this period, with a downregulation of factors involved in cell division and mitosis. The loss of *Cdk1* impaired CMBN but extended the neonatal CM proliferation window until day 10 post-birth. In adult hearts, the cardiac-specific ablation of Cdk1 triggered CM proliferation post-myocardial-infarction (MI) in specific zones, driven by the activation of *EGFR1* signaling and suppression of the anti-proliferative p38 and p53 signaling. This was accompanied by restoration of fractional shortening, mitochondrial function, and decreased reactive oxygen species. Additionally, cardiac hypertrophy was mitigated, and survival rates post-MI were increased in *Cdk1*-knockout mice. These findings reveal a novel role of *Cdk1* in regulating cell cycle exit and re-entry in differentiated CMs and offer insights into potential strategies for cardiac repair.

## 1. Introduction

Heart failure is the leading cause of morbidity and mortality in North America and the second leading cause of extended hospital stays [1]. The most common etiology of heart failure occurs following myocardial infarction (MI), underscoring the heart’s particular vulnerability to ischemic injury [2]. After an ischemic insult, the damaged cardiomyocytes (CMs) are replaced with fibrotic tissue [3]. This irreversible loss of CMs, coupled with the poor proliferative capacity of the surviving CMs, contributes to the development of heart failure [2]. Current treatment strategies primarily address the maladaptive neurohormonal changes but do not necessarily improve cardiac function [4]. Therefore, developing innovative approaches to restore cardiac function will not only improve patients’ quality of life but also reduce associated healthcare costs.

The regenerative capacity of the mammalian heart is lost shortly after birth when CMs transition from a proliferative to a quiescent state [5]. During the embryonic stage and up until birth, CMs undergo DNA replication, the G2/M phase, and cytokinesis, leading to CM proliferation [6,7,8,9]. Around postnatal day 5, CMs complete a final round of partial cell cycle activity, resulting in CMBN [9]. Thereafter, CMs exit the cell cycle and growth predominantly occurs through hypertrophy [6,7,8,10,11,12,13,14,15,16,17]. This transition is associated with changes in the levels of cell cycle regulators, where the expression of cell cycle inhibitors increases and cell-cycle-activating cyclin/Cdk complexes decrease [18,19,20,21,22,23,24,25,26]. Additionally, CM proliferation is tightly regulated by numerous intricate molecular pathways, including the Hippo/YAP mitogenic signaling pathway [11,27,28,29]. The progression through the cell cycle is positively regulated by Cdks and cyclins, their associated regulatory subunits [30], in a carefully orchestrated manner [31]. However, the factors controlling CM cell cycle progression during the postnatal period remain unknown.

*Cdk1*, also known as *Cdc2*, plays a central role in progressing the cell cycle. Cdk1 regulates essential processes during the G2/M phase transition and mitosis, ensuring accurate chromosome segregation and proliferation [32]. Cdk1 is activated by forming a complex with B-type cyclins in the late G2 phase [33]. In contrast, Wee1 kinase can phosphorylate and inhibit Cdk1, thereby delaying entry into mitosis [34]. Active Cdk1-cyclin B governs entry into mitosis by phosphorylating target proteins involved in chromatin condensation, nuclear envelope breakdown, and microtubule formation [33,35]. By controlling these events, Cdk1 ensures the proper execution of mitosis, safeguards the genome integrity, and maintains cell viability [36].

Based on these findings, we hypothesized that the loss of *Cdk1* in murine CMs would result in proliferative abnormalities in both neonatal and adult hearts. Here, we report the unexpected finding that *Cdk1* is required for normal cell cycle arrest and CMBN during the early postnatal period but is redundant for CM division. Furthermore, cardiac-specific *Cdk1* deletion in adult mice promoted cardiac repair through enhanced CM proliferation post-MI, driven by the activation of proliferative pathways such as *EGFR1* and the suppression of inhibitory pathways like p38 and p53. This highlights *Cdk1*’s critical role in regulating the balance between proliferation and differentiation in the heart.

## 2. Results

### 2.1. Cell Cycle Regulation of Cardiomyocytes during the Early Neonatal Period

**Figure 1:**
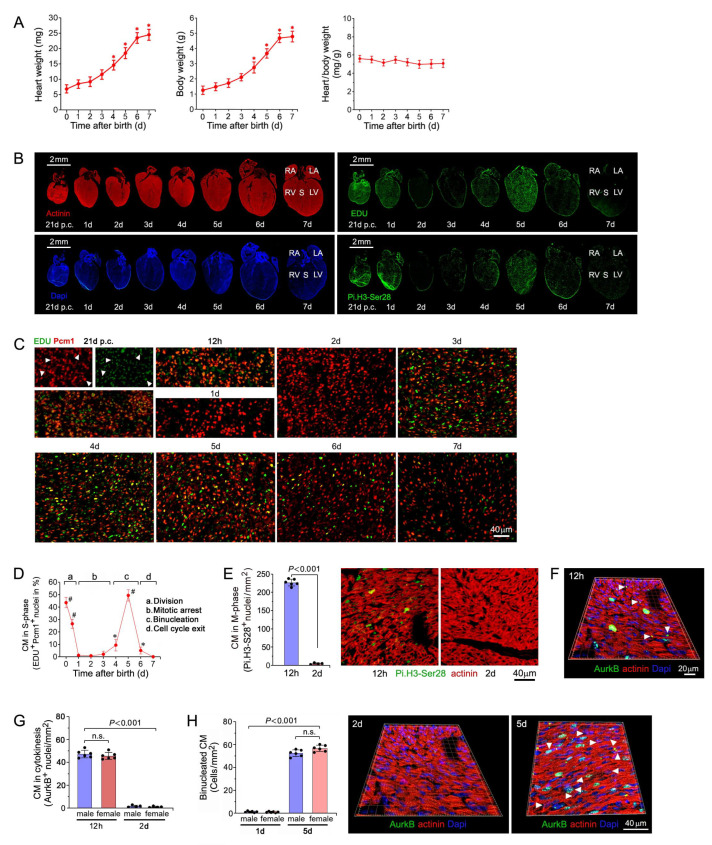
Cell cycle distribution and nucleation status of murine CMs during the first week after birth. (**A**) Heart weight, body weight, and heart/body weight ratios of neonatal mouse CMs during the first week after birth. Data are mean ± s.e.m. *n* = 6. * *p <* 0.01 vs. 4 d. (**B**) Wide-field immunofluorescence micrographs of neonatal mouse hearts. Tissue sections of whole hearts were fixed, permeabilized, and stained for EDU (green) to label S-phase nuclei or anti-Pi.H3-Ser28 (green) antibodies to detect mitotic nuclei and were co-stained with anti-sarcomeric alpha-actinin (red), a cytosolic CM-specific marker, and Dapi (blue) to visualize genomic DNA. RA, right atrium. LA, left atrium. RV, right ventricle. LV, left ventricle. S, interventricular septum. One representative result of 3 independent experiments is shown. (**C**) Wide-field immunofluorescence micrographs of CMs in the S phase in neonatal wt mice. Fixed specimens were co-stained for indirect immunofluorescence microscopy with S-phase marker EdU and with anti-Pcm1 antibodies, recognizing a CM-specific perinuclear marker. Arrow heads denote representative CM nuclei in the S phase. One representative result of 3 independent experiments is shown. (**D**) Quantification of neonatal CMs in the S phase. # *p* < 0.001 vs. 2 d. * *p <* 0.01 vs. 2 d. Data are mean ± s.e.m. *n* = 6. (**E**) Quantification (left panel) and confocal immunofluorescence microscopic analysis (right panel) of neonatal CMs in mitosis at 12 h and 2 d, employing antibodies to the Histone H3Pi-Ser28 (green) and cardiac actinin (red). Data are mean ± s.e.m. *n* = 6. (**F**) Analysis of neonatal CM undergoing cytokinesis at 12 h and 2 d. Three-dimensional (3D) reconstitution of typical confocal micrographs visualizes *AurkB*-positive midbody structure (white arrowheads) between 2 dividing daughter CMs. Fixed LV tissue sections were stained for indirect immunofluorescence microscopy analysis with antibodies to actinin (red), and *AurkB* (green). Specimens were co-stained with Dapi to detect genomic DNA. (**G**) CM cell division and exit from the cell cycle occurs in a sex-independent manner. Quantitative analysis of *AurkB*-positive neonatal CMs at 12 h and 2 d. n.s., not significant. Data are mean ± s.e.m. *n* = 6. (**H**) Quantification (left) and 3D reconstitution of typical confocal immunofluorescence microscopical analysis (right panel) of mono- vs. binucleated CMs in ventricular tissue sections. Cardiac specimens were stained with antibodies to *AurkB* (green) and actinin (red). Nuclear DNA was visualized employing Dapi. Data are mean ± s.e.m. *n* = 6.

We began our analysis with the assessment of heart weight/body weight (HBW) ratios in neonatal mice during the first 7 d after birth. We found that HBW did not significantly change over this period (Figure 1A). However, with the observation that mRNAs involved in DNA replication and mitosis showed a biphasic periodicity, we investigated DNA synthesis in CMs in the first 7 d after birth. Neonatal C57BL/6J wild-type (wt) mice were injected subcutaneously with a single dose of 5-ethynyl-2′-deoxyuridine (EDU), a thymidine analogue [37]. Cardiac specimens were prepared for indirect immunofluorescence microscopy employing an EDU assay, and anti-phospho-Histone Pi.H3-Ser28 (H3.Pi-S28) antibodies (Figure 1B) [38] to identify CMs in the S and M phases in the LV region, in conjunction with anti-cardiac actinin and 4′,6-diamidino-2-phenylindole (Dapi). We also co-stained neonatal cardiac sections with an antibody to pericentriolar material 1 (Pcm1), a CM-specific peri-nuclear marker [39,40], and EDU to specifically identify CMs in the S phase (Figure 1C). Using this approach, the CM labeling index was monitored from embryonic day 21 (0 d), neonatal 12 h, and 1 d through 7 d (Figure 1C). Exceedingly high labeling indexes were detected at 0 d and 12 h (Figure 1D). In contrast, levels of EDU/Pcm-1 double-positive CM nuclei dropped dramatically at 1 d (Figure 1D,E). This proliferative block was corroborated by immunofluorescence analysis of Pi.H3-Ser28-positive CMs in the M phase (Figure 1E), and aurora kinase B (*AurkB*)-positive CMs in cytokinesis (Figure 1F,G) [41]. Re-initiation of DNA synthesis occurred at 4 d, with a peak labeling index occurring at 5 d (49 ± 4.3%; *p <* 0.001 vs. 2 d). The number of EDU-positive CM nuclei decreased again markedly by 6 d (5.1 ± 2.1%; *p <* 0.01 vs. 2 d) (Figure 1D). There were no sex-dependent differences noted.

To ascertain that this second wave of DNA synthesis represented CMBN, we analyzed this morphological developmental change in 2-day- versus 5-day-old CMs by 3D reconstruction of confocal immunofluorescence micrographs of Dapi-stained LV cardiac sections [42]. CMBN was virtually absent in 1-day-old cardiomyocytes (Figure 1H). However, by day 5, 57 ± 9.1% of cardiomyocytes had undergone binucleation (*p* < 0.001 vs. 1 d). Combined, all these findings demonstrate that CM proliferation ceases in a sharply defined time window after birth and is followed by a distinct second phase of CMBN.

### 2.2. Differential mRNA Expression of Cell Cycle Factors in the Early Postnatal Period

**Figure 2:**
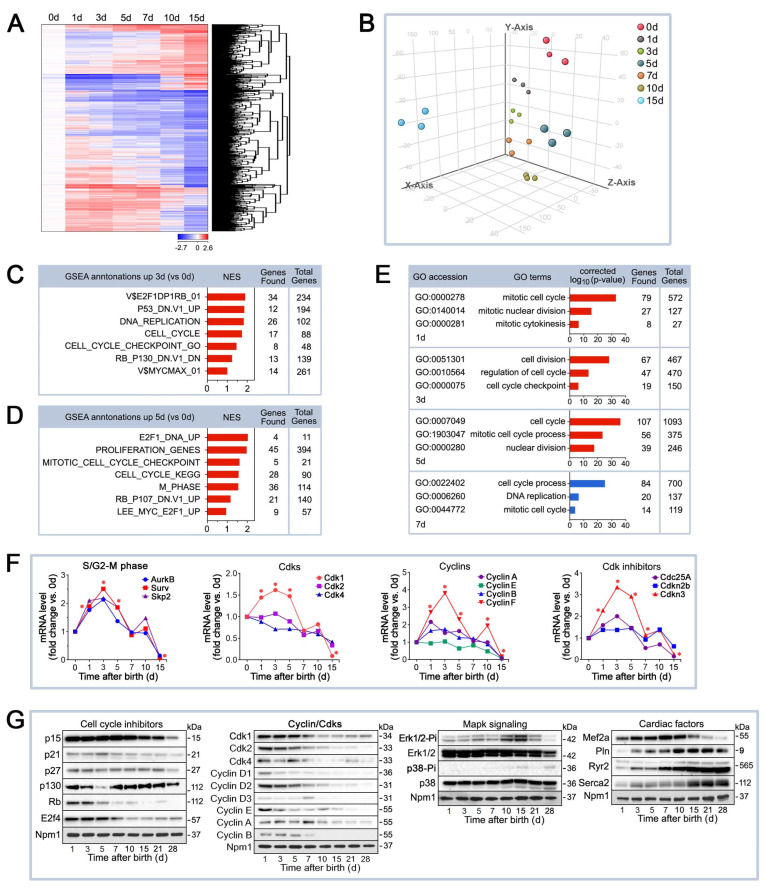
Genome-wide transcriptional changes in cell cycle factors in the early postnatal period. (**A**) Genome-wide transcriptional changes in the myocardium of neonatal mice. Heat map examining unsupervised hierarchical cluster analysis identified approximately 1900 of 43,000 individual genes (rows) that were enriched in the hearts of mice at 1 day to 15 days (columns), relative to controls at 0 days. Values (log2 expression) are shown by color and intensity of shading. Blue, repressed. Red, induced. *n* = 3 biological samples. Each biological sample consists of 12 hearts (0 d) to 3 hearts (15 d) serving as technical replicates. *p* < 0.01. Fold change cut-off 1.3. (**B**) Comparison of neonatal mouse transcriptomics by three-dimensional principal component analysis (3D-PCA). The transcriptome profiles are projected onto PC space. The 3D-PCA analysis demonstrates the presence of distinct developmental stages in neonatal mouse hearts during the early postnatal period. (**C,D**) Gene Set Enrichment Analysis (GSEA) of biological processes among all differentially expressed transcripts, assessed by over-representation of GSEA terms for the biological function of each transcript in neonatal mouse hearts at 3 d (**C**) and 5 d (**D**) vs. 0 d. NES, normalized enrichment scores. (**E**) GO term enrichment representing upregulated (red) and downregulated (blue) biological processes involved in the regulation of cellular proliferation in ventricular samples (vs. 0 d) from neonatal mice in the early postnatal period. (**F**) Significantly changed mRNA contents of cell cycle factors in murine hearts during the first 2 weeks after birth. RT-qPCR analysis to profile cell-cycle-related genes in neonatal mouse hearts. Data are mean ± s.e.m. * *p <* 0.01. *n* = 4. (**G**) Significantly changed protein levels of cell cycle regulators in murine neonatal hearts. Western blot analysis was carried out, employing specific antibodies as shown on the left of each panel. Immunoblots were repeated at least once, yielding similar results.

To elucidate the molecular mechanisms responsible for the cessation of proliferation, DNA binucleation, and cell cycle exit, we performed genome-wide mRNA microarray profiling of LV tissue samples [43]. Unsupervised hierarchical cluster analysis at a high confidence threshold revealed that approximately 1900 individual transcripts changed significantly in murine hearts at 1 d through 15 d, relative to 0 d (Figure 2A). Three-dimensional principal component analysis (3D-PCA) was performed to compare gene expression profiles across postnatal days, revealing clustering between the 1 d, 3 d, 7 d, and 10 d samples, and a closer association between the 0 d and 5 d samples (Figure 2B). This pattern is consistent with our microscopic results, which show CM proliferation at 0 d and CM binucleation at 5 d (Figure 1D). Both processes are largely absent in the other samples. While the clustering at 0 d and 5 d may not be as tight as the other groups, the 3D-PCA clearly indicates that these samples are visually distinct from the other time points, reflecting their unique biological characteristics. Moreover, the similarity between 0 d and 5 d can be explained by the fact that CM division and binucleation are regulated by a similar set of mRNAs, which may contribute to their closer positioning in the plot (Figure 2E).

Figure 1D shows that CM division was virtually absent between 1 and 3 days after birth. To identify gene clusters with similar biological functions in CM transcriptomes undergoing binucleation, we performed a Gene Set Enrichment Analysis (GSEA) on the transcriptomes of resting CMs at 3 d postnatal compared to 0 d, which represent proliferating CMs (Figure 2C). The top enriched gene sets in the 3 d vs. 0 d comparison include pathways associated with cell cycle control, DNA replication, and regulatory checkpoints (e.g., V$E2F1DP1RB_01, DNA_REPLICATION, CELL_CYCLE, and CELL_CYCLE_CHECKPOINT_GO). These data indicate that although CMs are largely resting at 3 d, there are still significant transcript levels of genes regulating DNA replication and the cell cycle. This may represent a transitional phase where the majority of CMs have exited the active proliferative phase (as seen at 0 d) but retain a baseline readiness for cell cycle re-entry. Gene sets such as “CELL_CYCLE_CHECKPOINT_GO” and “RB_P130_DN” support this negative control, likely through checkpoint mechanisms that prevent uncontrolled proliferation. Thus, the transcriptomic profile at 3 d likely reflects a transitional state where CMs are resting but remain poised to re-enter the cell cycle if needed.

Figure 2D shows the GSEA results comparing transcriptomes at 5 d vs. 0 d, where 5 d represents CMs undergoing binucleation. The top enriched GSEA gene sets in the 5 d vs. 0 d comparison include “E2F1_DNA_UP,” “PROLIFERATION_GENES,” “MITOTIC_CELL_CYCLE_CHECK POINT,” and “CELL_CYCLE_KEGG.” The GSEA signature at 5 d emphasizes that, even as binucleation occurs, the cell cycle is tightly regulated to ensure that CMs undergo binucleation rather than complete cytokinesis. While CMs have exited the typical division cycle, they retain essential regulatory mechanisms that guide the process of binucleation, a key step in the maturation of CMs.

Combined, at 3 d, the transcriptome reflects a resting phase with active cell cycle suppression, while at 5 d, the focus shifts towards binucleation, which involves the reactivation of distinct proliferative processes, yet these are tightly controlled to avoid cell division. Our view is further corroborated by a Gene Ontology (GO) analysis, which shows that the highest-ranked GO terms include transcripts involved in the regulation of nuclear division, negative regulation of cell division, and regulation of the mitotic cell cycle (Figure 2E).

Next, we validated the transcriptomic results of well-characterized cell cycle regulatory genes by qRT-qPCR analysis (Figure 2F) [42]. We found that the mRNA levels of a set of factors involved in the transition from the S phase into mitosis (*AurkB, Skp2*), G1-Cdks (*Cdk1, Cdk2, Cdk4*), G1/G2-cyclins (cyclins A, B, E, F), and key cell cycle activators (e.g., *Cdc25A*) and inhibitors (*Cdkn2b*, *Cdnkn3*) were significantly downregulated by postnatal day 7–10, coinciding with terminal cell cycle exit (Figure 2F). These analyses revealed that distinct cell cycle factors are differentially regulated in the immediate postnatal period. Our transcriptomic findings were confirmed by Western blotting of CM cell cycle inhibitors, cell cycle regulators, Mapk signaling pathways, and cardiac-specific genes (Figure 2G) [42]. Among all these factors, *Cdk1* was amongst the top ten enriched cell cycle genes that were selectively upregulated at 1 d and 5 d post-birth (Figure 2F). Thus, we decided to examine the potential role of *Cdk1* in the regulation of neonatal CM proliferation in vivo.

### 2.3. Ablation of Cdk1 Expands the Proliferative Time Window in Neonatal Cardiomyocytes

**Figure 3:**
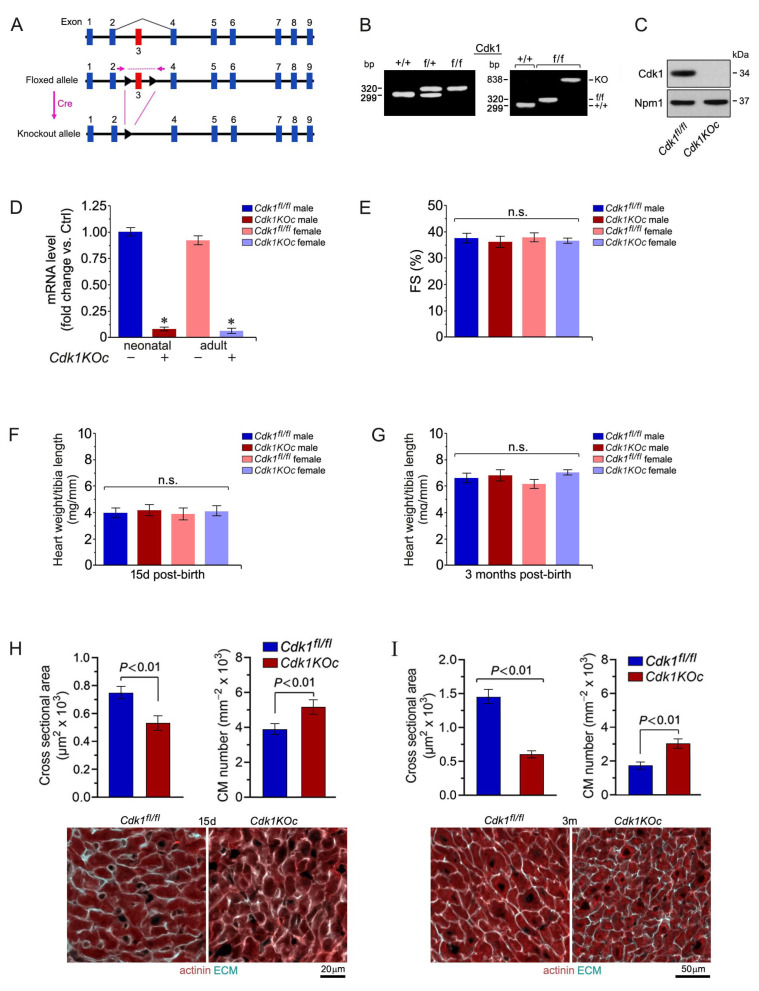
Normal heart function and tissue architecture in cardiac-specific *Cdk1KOc* mice. (**A**) Targeted *Cdk1* gene locus showing wild-type (top), floxed (middle), and deleted (bottom) alleles. (**B**) CM-specific knockout of *Cdk1*. PCR genotyping of genomic tail DNA from controls (left panel). PCR genotyping of DNA from *Cdk1KOc* hearts (right panel). (**C**) Western blot analysis of Cdk1 expression in LV extracts in neonatal CM isolated from 1-day-old *Cdk1KOc* mice employing anti-Cdk1 antibodies, as indicated on the left. To confirm equal loading, membranes were re-probed with anti-nucleophosmin (Npm1). Western blots were repeated twice with similar results. (**D**) Quantification of *Cdk1* mRNA levels in isolated neonatal CMs and in LV extracts of *Cdk1KOc* mice, as analyzed by RT-qPCR. Data are means ± s.e.m. *n* = 4. * *p* < 0.001 vs. controls. (**E**) Echocardiographic assessment (FS) of cardiac function in *Cdk1KOc* mice at 3 months of age. Data are mean ± s.e.m. *n* = 6. (**F**,**G**) Heart weight corrected for tibia length at 15 d after birth (**F**) and 3 months of age (**G**). Data are means ± s.e.m. *n* = 6. (**H,I**) Quantification (top panel) of cross-sectional area of CM at 15 d (**H**) and 3 months of age (**I**), as analyzed by confocal immunofluorescence microscopy (bottom panel). Data are means ± s.e.m. *n* = 6.

Factors that control cell cycle exit and binucleation in neonatal CMs are still not well characterized [44,45]. To investigate the mitotic regulators during this transition, we generated cardiac-specific *Cdk1*-mutant mice (Figure 3A). Transgenic mice homozygous for the loxP-flanked (floxed) allele of *Cdk1^fl/fl^* (controls) [46] were bred with αMyh6-Cre^+/+^ transgenic mice [47] to obtain *Cdk1^fl/fl^*;αMyh6 (*Cdk1KOc*) mice (Figure 3B). These mice had the *Cdk1* gene deleted specifically in CMs at embryonic day E14 post-coitum (p.c.), as analyzed by Western blotting and RT-qPCR (Figure 3C,D).

Cardiac function was not altered in *Cdk1KOc* mice versus controls, as assessed by echocardiographic measurement of fractional shortening (FS; *p >* 0.01) (Figure 3E). In growing animals, heart weight/tibia length (HTL) is a more reliable indicator of cardiac hypertrophy, especially in growing animals, as body weight can fluctuate due to factors unrelated to heart size. We found normal HTL ratios at 15 days post-birth for all four experimental groups (Figure 3F), demonstrating that the *Cdk1KOc* mice do not have a gross cardiac phenotype during the adolescent period. HTL ratios were also not significantly different in *Cdk1KOc* mice at 3 months of age relative to controls (*p >* 0.01) (Figure 3G). Intriguingly, we observed smaller CMs in *Cdk1KOc* animals compared to controls at 15 d (Figure 3H) and at 3 months post-birth (Figure 3H), suggesting that *Cdk1* may potentially regulate CM cell division during the early neonatal stage.

### 2.4. Loss of Cdk1 Prolongs the Proliferative Window of Cardiomyocytes during the Postnatal Stage

**Figure 4:**
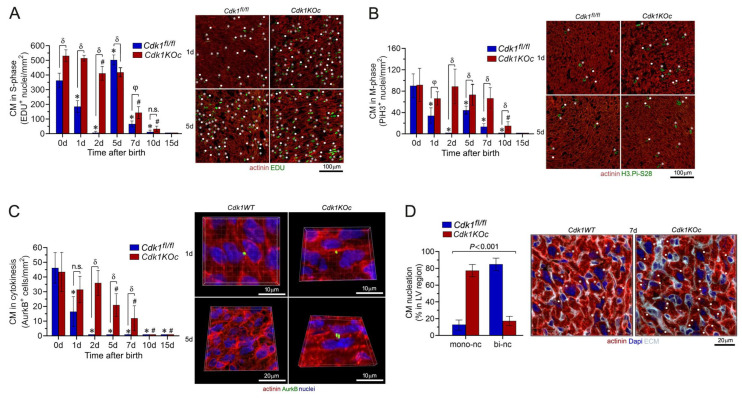
Cdk1 ablation increases neonatal cardiomyocyte proliferation in vivo. (**A**) Analysis of CMs in S phase in response to *Cdk1* ablation. Heart samples were collected from *Cdk1KOc mice* and controls. CMs were analyzed by confocal immunofluorescence microscopic employing belled the S-phase marker EDU (green) in conjunction with antibodies to CM-specific cytoplasmic marker α-actinin (red). Data are mean ± S.D. *n* = 4 biological replicates. * *p* < 0.01 vs. controls/0 d. ^#^ *p* < 0.01 vs. *Cdk1KOc*/0 d. ^δ^ *p* < 0.001. ^φ^ *p* < 0.01. n.s, not significant. (**B**) Analysis of CMs in M phase in *Cdk1KOc* mice. Fixed tissue sections were stained with antibodies to nuclear H3.Pi-S28 (green), an M-phase marker, in conjunction with α-actinin (red). Data are mean ± S.D. *n* = 4 biological replicates. * *p* < 0.01 vs. controls/0 d. ^#^ *p* < 0.01 vs. *Cdk1KOc*/0 d. ^δ^ *p* < 0.001. ^φ^ *p* < 0.01. (**C**) Analysis of CMs in cytokinesis in the absence of *Cdk1*. CMs were quantified by confocal immunofluorescence microscopic and 3D-reconstruction of serial confocal microphotographs by IMARIS software (version 10.2). Fixed tissue sections were co-immuno-stained for *AurkB*, a mid-body-specific cytokinesis marker, and α-actinin (red), in conjunction with DAPI to stain genomic DNA (blue). Data are mean ± S.D. *n* = 4 biological replicates. * *p* < 0.01 vs. controls/0 d. ^#^ *p* < 0.01 vs. *Cdk1KOc*/0 d. ^δ^ *p* < 0.001. ^φ^ *p* < 0.01. n.s, not significant. (**D**) CM-specific ablation of *Cdk1* decreases the ploidy of CMs. Quantification of CM nucleation in neonatal *Cdk1KOc* mice at 7 d post-birth (left panel) by confocal immunofluorescence microscopy and 3D reconstruction to distinguish mononucleated CMs from binucleated CMs (right panel). Fifty CMs located in 3 different regions were counted per sample. Data are means ± s.e.m. *n* = 6.

Initially, we investigated whether genetic silencing of *Cdk1* affects DNA synthesis in *Cdk1KOc* hearts in the early postnatal period. For analysis by confocal immunofluorescence microscopy, mice were injected subcutaneously with EDU, and hearts were excised, fixed, and sectioned. Subsequently, specimens were co-stained with antibodies to α-actinin, EDU, Pi.H3-Ser28, wheat germ agglutinin (WGA), and Dapi to visualize nuclear DNA. We detected a very high EDU-labeling index in CMs from *control* mice at 1 d that dropped dramatically at 2 d (*p* < 0.001 vs. 0 d) (Figure 4A, Figures S1 and S2). This was followed by a re-induction of DNA synthesis that peaked at 5 d. A progressive decrease in the number of CMs in the S phase was observed over 7 d and 10 d, until DNA synthesis in this cell type ceased completely by 15 d. Intriguingly, the numbers of EDU-positive CMs in *Cdk1KOc* mice remained markedly higher on 1 d, 2 d, and 5 d when compared to the controls (*p* < 0.001). Subsequently, DNA synthesis in *Cdk1*-deficient CMs progressively decreased over 7 d–10 d and was undetectable at 15 d (Figure 4A).

To analyze the distribution of CMs in mitosis, cardiac sections were co-stained with antibodies to H3.Pi-S28, an M-phase-specific nuclear marker, in combination with α-actinin immunostaining to identify CMs. Microscopic inspection showed dramatically elevated numbers of H3.Pi-S28-positive CMs in *control* animals at 0 d and 5 d (Figure 4B and Figure S3). This effect was very much lower in *control* CMs at 7 d and undetectable at 10 d and 15 d (*p* < 0.01 vs. 0 d). Again, the number of CMs in mitosis remained significantly higher in *Cdk1KOc* mice at between 1 d to 2 d and at 7 d post-birth in comparison to the *control* animals (*p* < 0.01). In this strain, H3.Pi-S28-positive CMs were never observed at 15 d (Figure 4B). Additionally, *Cdk1KOc* hearts at 4 w and 6 w of age failed to show any signs of cell cycle activity (Figure S4). Inspection of non-cardiomyocytes (NCMs) during the first week after birth revealed no significant differences in the number of cycling NCMs in *Cdk1KOc* compared to *control* hearts (Figure S5). Importantly, all these cell cycle events in CMs are sex-independent processes, since no difference was noted in the number of H3.Pi-S28-positive CMs in female mice in comparison to male mice (Figure S6).

Reportedly, CMs undergo a binucleation process at 5 d postnatally, implying that in these cells, the M phase can occur independently of cytokinesis [8]. Therefore, we analyzed whether *Cdk1*-mutant CMs completed cytokinesis by forming two daughter cells. Cytokinesis and polyploidization were distinguished by immunostaining with anti-AurkB antibodies to detect midbody structures between daughter CMs in fixed cardiac specimens by confocal microscopy. In the presence of *Cdk1*, CM cytokinesis was detected only at 0 d and 1 d post-birth based on the analysis of AurkB-positive midbody structures between CMs (*p* < 0.01 vs. 2 d) (Figure 4C). The second phase of DNA synthesis at 5 d was associated only with CMBN in *control* hearts, as indicated by the absence of *AurkB*-positive CM (*p* < 0.01 vs. 1d). In contrast, the ablation of *Cdk1* induced ongoing CM cytokinesis from 1 d to 7 d (*p* < 0.001 vs. 1 d–7 d controls) (Figure 4C). In particular, there was a significant increase in the percentage of binucleated CMs and a decrease in mononucleated CMs in *control* hearts at 7 d when compared to hearts from *Cdk1KOc* mice (*p* < 0.001) (Figure 4D). Thus, the loss of *Cdk1* not only extended the normal window of postnatal CM proliferation by 6 d but also markedly impaired CMBN. This effect was dependent on the absence of *Cdk1* and never observed in controls. Intriguingly, the high numbers of dividing CMs in the hearts of *Cdk1KOc* mice at 0 d are compatible with the concept that *Cdk1* is not absolutely required for prenatal CM proliferation.

### 2.5. Loss of Cdk1 Triggers Cell Cycle Re-Entry and Proliferation of Adult Cardiomyocytes Post-MI

**Figure 5:**
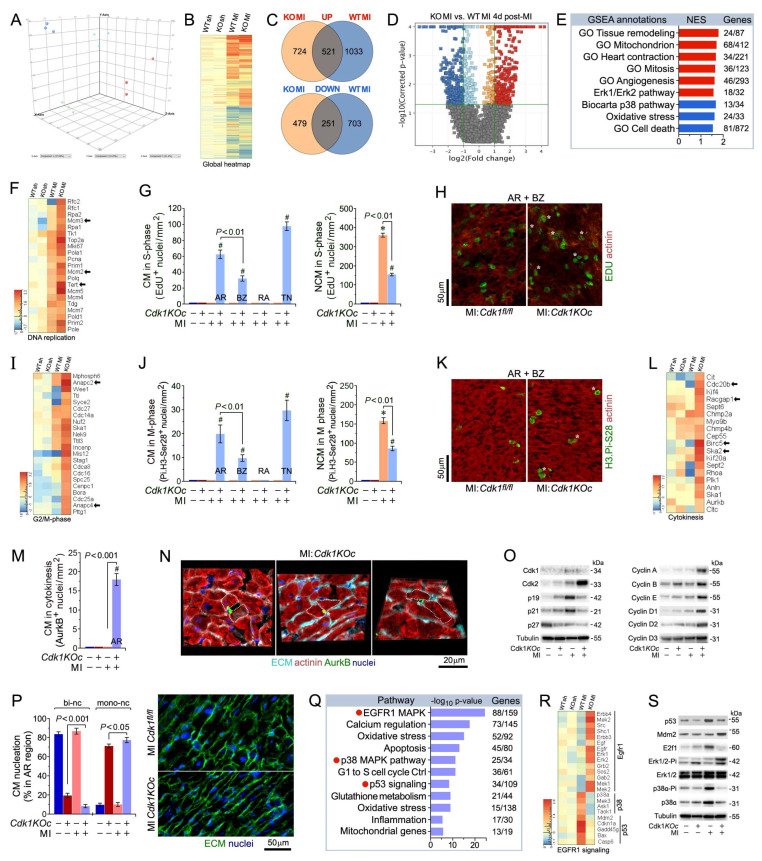
Cdk1 deletion induces proliferation of adult ventricular cardiomyocytes after myocardial infarction. (**A**) 3D-principal component analysis of ventricular transcriptomes as analyzed by RNA-seq at 4 d post-MI. (**B**) Heatmap of genome-wide differentially enriched ventricular transcriptomes in *Cdk1KOc* mice and controls at 4 d post-MI vs. sham. *n* = 3. Values (log2 expression) are shown by color and intensity of shading. Blue, repressed; red, induced. *n* = 3 biological replicates. *p* < 0.01. Fold change ± 2.0. (**C**) Venn diagram of differentially regulated genes in *Cdk1KOc* mice vs. controls at 4 d days post-MI. *n* = 3 biological replicates. *p* < 0.01. Fold change ± 2.0. (**D**) Volcano plot of RNA-seq results of transcripts selectively upregulated in *Cdk1KOc* mice post-MI vs. controls. (**E**) GSEA of different biological processes assessed by overrepresentation of GSEA terms for the biological function of each transcript in *Cdk1KOc* mice at 4 d post-MI. NES, normalized enrichment scores. (**F**) Heatmap of selectively enriched genes within the GO category of DNA replication, examining the impact of genomic modifications in *Cdk1*-deficient hearts (columns) on mRNA levels (rows). Arrows indicate key functional genes within this group. (**G**) Quantitative analysis of CMs and non-cardiomyocytes (NCMs) in S phase in *Cdk1KOc* mice at 4 d post-MI. Data are means ± s.e.m. *n* = 6. * *p* < 0.001 vs. sham/controls. ^#^ *p* < 0.001 vs. sham/ *Cdk1KOc*. (**H**) Confocal immunofluorescence microscopic analysis of S-phase CMs was carried out employing co-immunostaining of EDU (green) and anti-α-actinin (red). White asterisks, CM. (**I**) Heatmap of selectively enriched genes in the GO category G2/M phase. (**J**) Quantitative analysis of CMs and non-cardiomyocytes (NCMs) in M phase in *Cdk1KOc* mice at 4 d post-MI. Data are means ± s.e.m. *n* = 6. * *p* < 0.001 vs. sham/controls. ^#^ *p* < 0.001 vs. sham/ *Cdk1KOc*. AR, area at risk. BZ, border zone. RA, remote area. NCMs, non-cardiomyocytes. (**K**) Confocal immunofluorescence microscopic analysis of M-phase CMs was performed by confocal immunofluorescence microscopic analysis employing antibodies to the Histone H3Pi-Ser28 (green) and α-actinin (red). White asterisks, CM. Data are means ± s.e.m. *n* = 6. * *p* < 0.001 vs. sham/controls. ^#^ *p* < 0.001 vs. sham/*Cdk1KOc*. (**L**) Heatmap of selectively enriched genes in the GO category cytokinesis. (**M**) Quantitative analysis of CM in cytokinesis in *Cdk1KOc* mice at 4 d post-MI, as analyzed by 3D reconstitution of immunofluorescence micrographs. Data are means ± s.e.m. *n* = 6. ^#^ *p* < 0.001 vs. sham/ *Cdk1KOc*. (**N**) 3D reconstitution of immunofluorescence micrographs employing antibodies recognizing *AurkB*-positive midbody structures between 2 dividing daughter cells during cytokinesis. (**O**) Western blot analysis of cell-cycle factors total left-ventricular extracts using antibodies, as indicated on the left. Membranes were re-probed with anti-α-Tubulin for control of equal loading. Western blots were carried out twice employing two independent biological replicates yielding similar results. (**P**) Analysis of mono- and binucleated CMs in the AR at 21 d post-MI. Quantification of CM ploidy in adult *Cdk1KOc* mice (left) by confocal immunofluorescence microscopy and 3D reconstruction analysis (right). Serial confocal microphotographs were reconstructed into 3D employing IMARIS software to unambiguously distinguish mononucleated CMs from binucleated CMs. Fifty CMs located in 3 different regions in the AR/BZ zone were counted per specimen. Data are means ± s.e.m. *n* = 6. (**Q**) Pathway analysis based on differentially enriched mRNAs in *Cdk1KOc* mice relative to controls at 4 d post-MI. (**R**) Heatmap of selectively enriched transcripts within the top signaling pathways associated with CM proliferation, comparing *Cdk1KOc* and controls post-MI. (**S**) Immunoblot analysis of key genes in the *EGFR1*, p53, and p38α pathways.

To investigate the potential role of Cdk1 loss in CM proliferation and cardiac remodeling in a clinically relevant model of ischemic stress, 10–12-week-old male and female *Cdk1KOc* and *control* mice were subjected to MI by permanent ligation of the left anterior descending artery. Next, RNA-seq analysis on total left ventricular (LV) tissues from *Cdk1KOc* and *control* mice was performed at 4 days post-MI for genome-wide mRNA profiling. Our previous study identified the 4-day-post-MI time point as a critical ‘window’ that drives the proliferative cardiac phenotype in vivo [42]. Three-dimensional principal component analysis (3D-PCA) revealed distinct clustering of *Cdk1KOc* and *control* samples, both with and without MI, as analyzed using Strand-NGS software (version 4.1; Strand Life Sciences, Hebbal, Bangalore, India, Karnataka) (Figure 5A). Unsupervised hierarchical clustering at a high-confidence threshold identified 1433 transcripts that significantly differed between *Cdk1KOc* and *control* mice post-MI compared to sham-operated controls (Figure 5B,C). This finding was further supported by a Volcano plot analysis, which showed that *Cdk1* ablation led to the destabilization of mRNAs in *control* versus *Cdk1KOc* mice (Figure 5D).

To explore gene clusters with similar biological functions in mutant and wild-type transcriptomes, we performed a Gene Set Enrichment Analysis (GSEA) at 4 days post-MI treatment. We found that transcripts related to ventricular remodeling, mitochondrial function, cardiac contraction, and mitosis were among the most enriched GSEA terms (Figure 5E). Notably, transcripts for cell-cycle-promoting factors, such as *Mcm2, Mcm3*, and *Tert*, were selectively enriched at 4 days post-MI (Figure 5F). At 4 d post-MI, the area at risk (AR) in *Cdk1KOc* and *control* groups was of equal size and involved greater than 80% of the cross-sectional area of the left ventricle, measured at the level of the papillary muscles (Figure S7). We also noted a decreased sarcomeric α-actinin expression and lower extracellular matrix content, as evaluated by wheat germ agglutinin (WGA) staining. The border zone (BZ) showed α-actinin expression comparable to that of the area at risk, with a markedly higher level of WGA reactivity. This is in stark contrast to the remote area (RA) where both α-actinin and WGA are observed at levels comparable to sham *Cdk1KOc* and sham controls (Figures S7 and S8).

To further define the role of Cdk1 loss in cardiac remodeling after an MI, DNA synthesis was assessed using EdU labeling in adult mice, followed by confocal immunofluorescence microscopy. Intriguingly, we observed the following numbers of S-phase CMs in the LV region of *Cdk1KOc* mice at 4 d post-MI: (1) AR 61 ± 10.9 nuclei/mm^2^, (2) BZ 33 ± 7.6 CM nuclei/mm^2^, (3) RA 1.2 ± 3.4 CM nuclei/mm^2^, and (4) total number (TN) 98 ± 11.3 CM nuclei/mm^2^ (*p* < 0.01) (Figure 5G,H, Figures S7 and S8). These results clearly demonstrate that the induction of DNA synthesis in CMs lacking *Cdk1* requires an ischemic stress.

Assessment of numbers of CMs in the LV region of *Cdk1KOc* mice in the M phase by immunofluorescence microscopy employing anti-phospho-Histone 3 phosphorylated at H3.Pi-S28 showed (1) AR 19.8 ± 7.6 CM nuclei/mm^2^, (2) BZ 9.7 ± 3.2 CM nuclei/mm^2^, (3) RA zero CM nuclei/mm^2^, and (4) TN 30 ± 8.6 CM nuclei/mm^2^ (*p* < 0.01) (Figure 5I–K and Figure S9). In all these experiments, EDU-positive and H3.Pi-S28-positive CMs were absent from the *control* mice. In addition, the induction of CMs in the M phase post-MI was restricted to CMs residing in the BZ and was absent from the right ventricle.

Next, we analyzed whether CMs in the LV region of *Cdk1KOc* mice completed cytokinesis by dividing into two daughter cells by detecting midbody structures in the final phase of daughter cell separation employing anti-AurkB antibodies. MI in *Cdk1KOc* animals induced cytokinesis in 15.2 ± 4.6 CM/mm^2^ in the AR and minimally in the BZ (*p* < 0.001) (Figure 5L–N and Figure S10) that was not observed in the controls. This supports the notion that *Cdk1* deletion restricts proliferation to a specific subpopulation located in an ischemic milieu. Of note, CM proliferation in hearts from sham *Cdk1KOc* and sham *control* mice was never observed.

Infarct ‘wound healing’ is characterized by the initiation of non-CM (NCM) proliferation that mainly comprises endothelial cells and fibroblasts [48]. We noticed a significant increase in the numbers of NCMs in the S and M phases in the LV region of *Cdk1KOc* and *control* mice at 4 d following MI (*p* < 0.001 vs. sham *Cdk1KOc* and sham controls) (Figure 5G,J and Figure S7). The number of proliferating EDU-positive NCMs was markedly lower in the hearts of *Cdk1KOc* mice compared to the controls (150 ± 6.1 vs. 351 ± 9.6 NCM nuclei/mm^2^). All these findings support the view that *Cdk1* ablation induces adult CM proliferation after ischemic injury. We and others have previously shown that cell cycle re-entry in adult CMs is tightly regulated by *Cdk2* [42]. At 4 d post-MI, sham control hearts lacked an induction of Cdk2 protein relative to *Cdk1KOc* post-MI (Figure 5O). Elevated *Cdk2* protein levels were accompanied by the sustained induction of proliferative cyclins A, B, D2, and E (Figure 5O). Intriguingly, ablation of *Cdk1* also led to extremely low protein levels of *Cdk2*-inhibitory p21 and p19, cdk4/6 inhibitors.

We have established that adult *Cdk1KOc* mice have developed a hypertrophic heart (Figure 3I) that mainly consists of mononucleated CMs (78 ± 7.6%; *p* < 0.001 vs. *control*) (Figure 4D). Thus, we examined whether *Cdk1* deficiency can further influence the ploidy of CMs in the LV region post-MI. We observed a small but significant increase (11%) in mononucleated CMs, and a decrease (−75.4%) in binucleated CMs in the AR/BZ areas in *Cdk1KOc* mice post-MI (*p* < 0.001) (Figure 5P). This data provides additional evidence that genetic *Cdk1* ablation leads to CM proliferation rather than polyploidy.

### 2.6. Activation of EGFR1 Signaling Promotes Cardiomyocyte Proliferation in Cdk1KOc Mice Post-MI

RNA-seq analysis of LV tissue samples at 4 d post-MI identified differential pathway activation between *Cdk1KOc* and *control* mice, as analyzed by employing the pathway analysis module of Strand-NGS (Figure 5Q). The *EGFR1* signaling pathway was significantly upregulated in the *Cdk1KOc* MI group, as indicated by the enrichment of 88 out of 159 genes within this pathway (*p* < 0.001). This activation is associated with enhanced CM proliferation in the *Cdk1KOc* mice post-MI. In contrast, the p53- and p38-MAPK signaling pathways were predominantly activated in the *control* group. The activation of these pathways is known to inhibit CM division, suggesting that *control* mice respond to MI with signals that block cell cycle re-entry [49,50]. In contrast, the absence of Cdk1 allows for the activation of proliferative pathways, such as *EGFR1*, potentially facilitating CM proliferation (Figure 5Q) [51]. The heatmap in Figure 5R further corroborates the pathway analysis by displaying the expression profiles of key genes involved in the EGFR1, p38, and p53 signaling pathways across the four different experimental groups. Notably, key genes involved in the EGFR1 pathway, such as *ErbB4*, *Erk1*, and *Sos2*, show marked upregulation in the *Cdk1KOc* MI group when compared to the controls, aligning with the observed pathway activation. Conversely, key genes associated with the p38-MAPK and p53 pathways, including p38α, *Cdkn1*, and *Gadd45g*, are upregulated in the control group post-MI, which is consistent with the inhibition of CM proliferation in this cohort (Figure 5R). Western blot analysis confirms the activation of these pathways at the protein level (Figure 5S). In *Cdk1KOc* mice post-MI, there is an increase in phosphorylated ERK1/2 (Pi-ERK1/2), a key downstream effector of EGFR1 signaling, supporting the role of this pathway in promoting CM proliferation. In contrast, control mice show increased levels of p53 and phosphorylated p38α (Pi-38α), further validating the RNA-seq findings that these pathways are involved in blocking CM division following an MI. The presence of these protein markers in the *Cdk1KOc* and *control* groups provides strong evidence for the differential activation of proliferative vs. inhibitory pathways depending on the genetic background of the experimental strains.

### 2.7. Cdk1 Deficiency Mitigates Hypertrophy, Boosts Survival, and Improves Heart Function Post-MI

**Figure 6:**
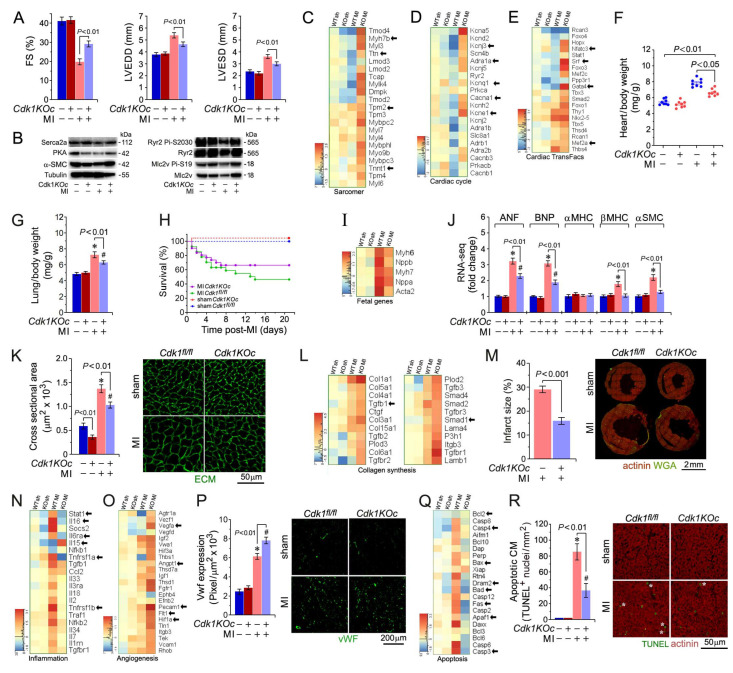
Cdk1 ablation normalizes contractility with improved survival post-MI. (**A**) Echocardiographic assessment of cardiac function. FS, LVEDD, and LVESD in *Cdk1KOc* mice at 21 d post-MI. Data are mean ± s.e.m. *n* = 6. (**B**) Immunoblot analysis of key sarcomeric and calcium-handling proteins in *Cdk1KOc* mice and controls at 21 d post-MI employing specific antibodies, as shown on the left. Membranes were re-probed with anti-Tubulin for control of equal loading. Western blots were carried out twice, employing two independent biological replicates, yielding similar results. (**C**–**E**) Heatmap analysis of transcript levels in key cardiac Gene Ontology terms: Sarcomeric structure (**C**), cardiac cycle (**D**), and cardiac transcription factors (Cardiac TransFacs) (**E**) at 4 d post-MI. (**F**) Heart weight corrected for body weight at 21 d post-MI. Data are means ± s.e.m. *n* = 6. (**G**) Lung weight corrected for body weight at 21 d post-MI. Data are means ± s.e.m. *n* = 6. * *p* < 0.01 vs. sham controls. * *p* < 0.01 vs. sham *Cdk1KOc*. (**H**) Mantel–Cox test shows improved survival in *Cdk1KOc* mice compared to controls at 21 d post-MI (*p* < 0.001). MI *Cdk1KO n* = 20; MI controls *n* = 24; sham *Cdk1KO n* = 6; sham controls *n* = 6. (**I**) Heat map examining the impact of genomic modifications in *Cdk1*-deficient hearts (columns) on transcript levels of the fetal gene program (rows) at 4 d post-MI. (**J**) RNA-seq quantification of mRNA levels for hypertrophic marker genes at 21 days post-MI. Data are presented as mean ± s.e.m., *n* = 3. * *p* < 0.01 vs. sham controls. ^#^ *p* < 0.01 vs. sham *Cdk1KOc*. (**K**) Quantification of cross-sectional area of CM in the border zone at 21 d post-MI (left), as analyzed by confocal immunofluorescence microscopy (right). Data are means ± s.e.m. *n* = 6. * *p* < 0.01 vs. sham controls. ^#^ *p* < 0.01 vs. sham *Cdk1KOc*. (**L**) Heat maps examining protein coding transcripts important for the regulation of collagen synthesis at 4 d post-MI. (**M**) Quantification of infarct sizes at 21 d post-MI (left panel). Representative confocal immunofluorescence micrographs of WGA-stained cardiac cross sections recognizing fibrotic ECM (right). Data are means ± s.e.m. *n* = 6. (**N**,**O**) Heatmap analysis of gene expression in key cardiac Gene Ontology terms: Inflammation (**N**), and angiogenesis (**O**) at 4 d post-MI. (**P**) Quantification of capillary density at 4 d post-MI (left), as determined in myocardial LV cross sections employing anti-Von-Willebrand-factor staining (right; green). Data are means ± s.e.m. *n* = 6. * *p* < 0.01 vs. sham controls. ^#^ *p* < 0.01 vs. sham *Cdk1KOc*. (**Q**) Heat map examining the impact of *Cdk1* ablation on validated pro-apoptotic transcript levels. (**R**) Quantification of CM apoptosis (left) in LV sections (right) at 2 d post-MI. White stars, CMs. Data are means ± s.e.m. *n* = 6. * *p* < 0.01 vs. sham controls. ^#^ *p* < 0.01 vs. sham *Cdk1KOc*.

Next, we assessed the physiological consequences of *Cdk1* loss by echocardiographic measurements of fractional shortening (FS), left ventricular end-diastolic diameter (LVEDD), and left ventricular end-systolic diameter (LVESD) across *Cdk1KOc* and controls at 21 d post-MI (Figure 6A). FS, an important indicator of systolic function, was similar between sham-operated controls and *Cdk1KOc* mice. However, FS in *Cdk1KOc* mice increased by 49% relative to the controls (*Cdk1KOc* post-MI: 29 ± 2%, vs. controls post-MI: 20 ± 3%; *p* < 0.01) (Figure 6A). These data demonstrate that *Cdk1* ablation preserved contractile function post-MI, which is crucial for limiting the progression to heart failure. We observed that both LVEDD and LVESD, parameters reflecting LV size and remodeling, were significantly improved in *Cdk1KOc* mice against the controls post-MI. LVEDD was significantly lower in *Cdk1KOc* mice (4.8 ± 0.4 mm) compared to the controls (5.3 ± 0.5 mm; *p* < 0.01), indicating less ventricular dilatation (Figure 6A). Similarly, LVESD was reduced in the *Cdk1KOc* group (3.0 ± 0.2 mm) next to the controls (3.8 ± 0.3 mm, *p* < 0.01). These findings show that *Cdk1* ablation attenuates adverse remodeling post-MI, implying that abrogation of *Cdk1* function has cardioprotective effects.

Western blot analysis of LV tissue samples in Figure 6B shows several key cardiac proteins, including Serca2a and RyR2, along with their phosphorylated activated forms. The *Cdk1KOc* cohort demonstrates altered protein expression post-MI that correlates with improved calcium handling and contractility when compared to the controls. Figure 6C shows levels of sarcomeric genes, which are crucial for cardiac muscle contraction, across different experimental groups. Notably, key genes such as *Ttn, Myh7b,* and *Tnnt1* are significantly upregulated in the *Cdk1KOc* MI group relative to the controls. This elevated expression of sarcomeric genes in *Cdk1KOc* mice post-MI suggests enhanced contractile machinery, contributing to improved cardiac function. The heatmap in Figure 6D shows the differential expression of genes involved in the cardiac cycle. Key genes such as *Kcnj3, Scn4b,* and *Prkca*, which are involved in cardiac rhythm and contraction regulation, are specifically upregulated in *Cdk1KOc* mice post-MI. This upregulation implies that the *Cdk1* knockout promotes a more efficient cardiac cycle post-MI, likely contributing to the observed preservation of heart function in these mice.

Figure 6E illustrates the significant upregulation of transcript levels of various cardiac transcription factors, highlighting *Gata4, Srf*, and *Nkx2-5* in *Cdk1KOc* mice post-MI compared to the controls. These transcription factors are known to regulate genes critical for cardiac function and remodeling, and their increased expression in the knockout mice may play a pivotal role in the observed enhancement of cardiac function post-MI. Collectively, these findings indicate that the upregulation of sarcomeric and cardiac cycle genes, along with key transcription factors, contributes to the superior cardiac function observed in *Cdk1KOc* mice post-MI.

Increases in HBW ratios in *Cdk1KOc* mice were significantly less post-MI versus MI/controls (Figure 6F). Moreover, lung weight/body weight (LBW) ratios, an index of pulmonary congestion, were also less elevated in *Cdk1KOc* mice as opposed to controls post-MI (Figure 6G). Importantly, the Mantel–Cox test showed significantly improved survival in *Cdk1KOc* mice at 21 d post-MI relative to the controls (*p* < 0.001) (Figure 6H). RT-qPCR analysis of canonical hypertrophic marker genes in LV tissue samples revealed decreased transcript levels ANP, BNP, and β-MHC in *Cdk1KOc* mice post-MI against controls (*p <* 0.01) at 3 d and 21 d (Figure 6I,J)). Moreover, the CM cross-sectional area (Figure 6K) and infarct size (Figure 6L,M), as measured at the peri-infarct zone, were significantly lower in *Cdk1KOc* mice in contrast to the controls post-MI (*p <* 0.01).

Inflammation typically precedes angiogenesis in the sequence of biological responses following MI [3,4,52]. The heatmap in Figure 6N shows the expression levels of inflammation-related genes across different experimental groups. In LV tissue samples of *Cdk1KOc* mice post-MI, several key pro-inflammatory genes (*Stat1, Il6ra, Nfkb1, Tgfbr1*) are significantly upregulated against the *control* cohort. This indicates a heightened inflammatory response in *Cdk1KOc* mice following MI. Notably, genes involved in promoting angiogenesis (*Vegfa, Flt1, Pecam1, Angpt1*) are upregulated in the *Cdk1KOc* mice relative to the controls post-MI (Figure 6O). This suggests that *Cdk1KOc* mice may have enhanced angiogenic responses post-MI, potentially contributing to better tissue repair and remodeling. Additionally, immunofluorescence images of fixed *Cdk1KOc*-derived cardiac tissue samples exhibit a significantly higher vWF expression post-MI (*p* < 0.01) versus controls, indicating increased endothelial cell activity and angiogenesis (Figure 6P). All these findings indicate a higher degree of coronary angiogenesis in *Cdk1KOc* mice post-MI as opposed to their wt counterparts.

Our heatmap analysis reveals that key apoptotic genes (*Bax, Casp3, Fas*, and *Bcl2*) are upregulated in LV tissue samples of the controls in comparison to *Cdk1KOc* animals post-MI (Figure 6Q). Quantification of apoptotic CM through TUNEL staining and fluorescence microscopic analysis demonstrates a significantly higher number of apoptotic CMs in the controls versus *Cdk1KOc* mice post-MI (*p* < 0.01) (Figure 6R), suggesting that Cdk1 deletion protects against CM apoptosis post-MI.

### 2.8. Cdk1 Loss Preserves Mitochondrial Energetics by Protecting against Ischemic Oxidative Stress

**Figure 7:**
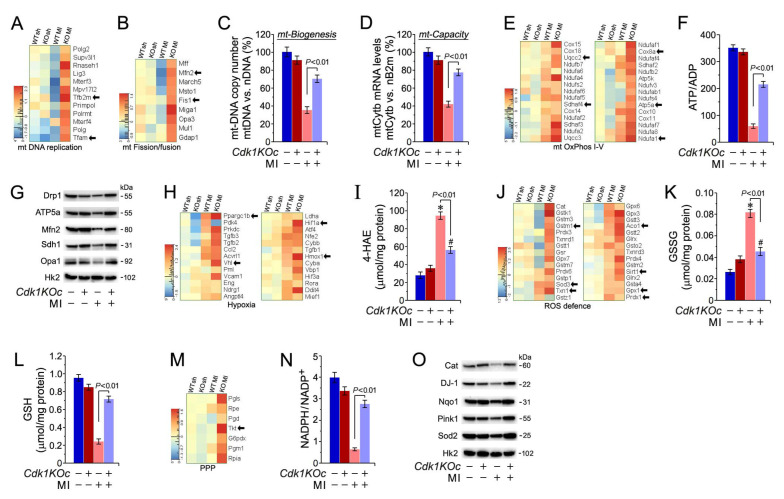
Cdk1 deficiency inhibits cardiac oxidative stress and improves mitochondrial ATP production. (**A**,**B**) Heat map examining the impact of Cdk1 loss on mRNA levels of mt-DNA replication (**A**) and mt fission/fusion (**B**) at 4 d post-MI. (**C,D**) Mt biogenesis (**C**), defined as relative DNA copy number of mt. Encoded *Cytb* gene normalized to the copy number of the nuclear gene *B2m*, was determined by qPCR. Mt capacity, defined as relative mRNA (**D**) levels of the nuclear gene cytochrome b (*Cytb*), a constituent of OxPhos complex III, normalized to *B2m* transcript expression, was determined by RT-qPCR at 4 d post-MI. Data are means ± s.e.m. *n* = 4. (**E**) Heat maps examining the impact of *Cdk1* deficiency on components of mt oxidative phosphorylation. (**F**) Quantification of ATP/ADP ratios in *Cdk1KOc* mice vs. controls at 4d post-MI. Data are means ± s.e.m. *n* = 4. (**G**) Immunoblot analysis of proteins in *Cdk1KOc* mice important for the mt electron transport chain (*ATP5a*, complex V; *Sdh1*, complex II) and key factors necessary for mt fusion/fission (*Drp1, Mfn2, Opa1*) at 4 d post-MI employing specific antibodies, as shown on the left. Membranes were re-probed with anti-hexokinase 2 (*Hk2*) for control of equal loading. Western blots were carried out twice employing two independent biological replicates yielding similar results. (**H**) Heat maps investigating the consequences of *Cdk1* ablation on hypoxia-related gene expression. (**I**) Levels of 4-HAE, a biomarker for oxidative lipid damage, in hearts of *Cdkn1KOc* mice at 4 d post-MI. Data are means ± s.e.m. *n* = 4. * *p* < 0.01 vs. sham controls. ^#^ *p* < 0.01 vs. sham *Cdk1KOc*. (**J**) Heat maps showing enrichment of induced (red) and repressed (blue) protein coding gene transcripts involved in the regulation of the GO term “reactive oxygen species (ROS) defence”. (**K**,**L**) Levels of GSSG (K) and GSH (L), indicators of cardiac oxidative stress, in *Cdk1KOc* mice in vs. controls at 4 d post-MI. Data are means ± s.e.m. *n* = 4. * *p* < 0.001 vs. sham *control*. (**M**) Heat maps showing enrichment of induced (red) and repressed (blue) protein coding gene transcripts involved in the regulation of the pentose phosphate pathway (PPP). (**N**) Levels of NAPDH, an important metabolite in GSH synthesis, in *Cdk1KOc* mice in vs. controls at 4 d post-MI. Data are means ± s.e.m. *n* = 4. (**O**) Immunoblot analysis of important anti-oxidative factors for *Cdk1KOc* mice and controls at 4 d post-MI, employing specific antibodies, as shown on the left. Membranes were re-probed with *Hk2* antibodies for loading control. Western blots were carried out twice employing two independent biological replicates giving similar results.

We have previously shown that MI-induced oxidative stress decreases in ATP levels negatively impact the contractile function and survival of CMs [42]. Thus, we analyzed gene expression of mt-DNA replication and mt-dynamics in LV samples of *Cdk1KOc* mice post-MI. The heatmap in Figure 7A shows that the transcript levels of key genes involved in mt-DNA replication such as *Tfam, PrimPol,* and *Tfb2m* are upregulated in *Cdk1KOc* mice post-MI. Notably, the expression of genes related to mt fission and fusion like *Fis1, Mfn2*, and *Mff* are also significantly higher in the *Cdk1KOc* mice post-MI (Figure 7B). Next, the mt-DNA copy number was quantified by qPCR using mt-Cytb that were normalized to the nuclear-encoded gene *B2m*. In *Cdk1KOc* mice post-MI, we found a significant restoration of the mt-DNA copy number (*p* < 0.01) (Figure 7C) in contrast to the controls. This restoration was accompanied by an increase in mitochondrial respiratory capacity (*p* < 0.01) (Figure 7D) and notably higher ATP/ADP ratios in the myocardium of *Cdk1KOc* mice vs. controls (*p* < 0.01) (Figure 7E–G).

Moreover, we also observed significantly decreased levels of cytotoxic 4-HAE, an indicator of ROS-dependent lipid peroxidation [27] (Figure 7H,I) with upregulated glutathione/oxidized glutathione (GSH/GSSG) ratios in LV samples of *Cdk1KOc* mice post-MI (Figure 7J–L). The heatmap in Figure 7M illustrates transcript levels of genes involved in the pentose phosphate pathway (PPP), which is critical for generating glutathione, a key molecule for producing NADPH to combat oxidative stress [53]. In *Cdk1KOc* mice post-MI, there is an upregulation of genes such as *Tkt* and *G6pdx,* indicating increased PPP activity compared to controls. This upregulation correlates with higher NADPH/NADP+ ratios in *Cdk1KOc* hearts subjected to MI (Figure 7N) and implies an enhanced capacity to neutralize oxidative stress (*p* < 0.01). This view agrees with our Western blot analysis showing elevated levels of antioxidative enzymes such as *Sod2, Nqo1*, and *Pink1* in *Cdk1KOc* mice post-MI, underscoring the improved oxidative stress response associated with *Cdk1* knockout (Figure 7O).

## 3. Discussion

*Cdk1* has a distinct role in embryonic mammalian proliferation that is not fulfilled by *Cdk2/4/6* [54]. Yet, there is a paucity of experimental evidence regarding the role of *Cdk1* in regulating CM proliferation during the neonatal phase and post-MI. Our data demonstrate that Cdk1 activity is crucial for CM cell cycle arrest at 1 d and CMBN at 5 d after birth. Notably, the ablation of *Cdk1* causes CMs to remain in a proliferative state until 10 d. Additionally, the genetic ablation of *Cdk1* promotes CM proliferation and aids in cardiac repair after MI in the murine adult heart. This study is the first to reveal *Cdk1*’s dual roles in CMM.

It was surprising to discover that Cdk1 intrinsically inhibits the proliferation of both neonatal and adult CMs post-MI. This finding contradicts the prevailing belief that Cdk1 is necessary for cell proliferation. While germline *Cdk1KO* mice do not progress beyond the two-cell embryonic stage [54], it is interesting that *Cdk1* can offset the lack of individual Cdks in *Cdk2/4/6* triple-KO embryos, allowing them to develop normally [55,56]. The triple-KO embryos maintain Cdk1 expression, supporting regular cell proliferation until mid-gestation. Interestingly, the introduction of *Cdk2* into the *Cdk1* gene locus in mice did not prevent early embryonic death. In our study, *Cdk1KOc* mice were born at expected Mendelian ratios and exhibited hypertrophic ventricular walls but maintained normal cardiac function.

The rate of CM proliferation in mouse hearts varies between embryonic and postnatal stages. The four-chambered mammalian mouse heart is completely developed by embryonic day E12.5 [57]. In mice, CM proliferation peaks around E10-12 and then gradually diminishes until birth. This pattern might shed light on our observation that Cdk1 is not essential for CM proliferation after day E14, which is after the initiation of α-MHC Cre-dependent homologous recombination, and during the early postnatal phase. Rather, we hypothesize that Cdk1 is primarily required during the initial phases of heart formation in embryogenesis. Our data further corroborate the notion that *Cdk2/4/6*, either individually or in conjunction, can offset the absence of Cdk1 activity during the cell cycle progression of fetal and neonatal CMs. Comparable to *Cdk1KOc* mice, transgenic mice with cardiac-specific overexpression of Cdk2 in adults displayed an increase in the number of smaller, mononucleated CMs, suggesting enhanced CM division [58]. Hearts with Cdk2 overexpression exhibited normal FS, even with elevated levels of the canonical hypertrophic marker genes ANF and β-MHC. In contrast, the triple-KO mice lacking cyclin D1/2/3 present hypoplastic ventricular walls accompanied by severe anemia. These animals typically die around mid- to late gestation around embryonic day E16.5 [59].

Our study revealed that the loss of *Cdk1* post-MI leads to the activation of the *EGFR1* signaling pathway, which promotes CM proliferation [51] and the suppression of the p38 [50] and p53 pathways [49]. This is associated with blocking CM division, thereby allowing for an extended proliferative window in *Cdk1KOc* mice. This shift in signaling pathways is crucial for enhancing cardiac repair and preserving cardiac function post-MI. Furthermore, Cdk1 ablation was found to significantly enhance mitochondrial function and reduce the production of ROS, as evidenced by the upregulation of mitochondrial biogenesis and antioxidative enzymes in the *Cdk1KOc* mice. This improvement in mitochondrial energetics likely contributes to the preserved cardiac function observed in these mice.

We also demonstrate that *Cdk1* is important for the regulation of the CM division cycle early after birth. Unexpectedly, the genetic deletion of *Cdk1* in neonatal mouse CMs effectively disabled the intrinsic proliferative block at 1 d post-birth, extending the postnatal proliferative window to 10 days. However, there are still gaps in our understanding of the mechanisms that govern CMBN [60,61]. In inbred C57BL/6 mice, commonly employed for cardiological studies, 8-10% of CMs are mononucleated [60]. Notably, non-epigenetic mechanisms such as certain polymorphisms located in X-linked and autosomal genes can influence the outcome of mitosis and karyokinesis in a nonautonomous-CM fashion [60]. Our findings found no evidence to support this view during adolescent murine growth, as the HTW ratio of *Cdk1KOc* mice at 15 d and 3 m did not significantly alter from the controls (Figure 3F,G). In addition, a proliferative burst at 15 d post-birth has been suggested to give rise to binucleated mouse CMs [62]. This event is mediated by the thyroid hormone–IGF-1-Akt signaling pathway. However, several independent studies, including our present work, were unable to confirm this preadolescent event [63,64].

## 4. Conclusions

In this study, we investigated the regulation of the cell division cycle in CMs during the early neonatal period and its impact on heart regeneration following an MI. Our results revealed a tightly controlled sequence of events: CM proliferation ceases shortly after birth, followed by a distinct phase of CMBN. *Cdk1* plays a pivotal role in regulating both CMBN and proliferation during this period. The loss of *Cdk1* extended the neonatal proliferative window, increasing the number of dividing CMs. In adult mice, *Cdk1* ablation post-MI activated the *EGFR1* signaling pathway, promoting CM proliferation, while downregulating the p38 and p53 pathways, which are typically antiproliferative. This was associated with restoration of FS, enhanced mt function, and a reduction in oxidative stress post-MI.

*Cdk1* emerges as a potential key regulator of heart regeneration post-MI. Targeting Cdk1-related pathways holds significant therapeutic potential for enhancing heart regeneration after an MI. Future research should explore the interplay between *Cdk1* and other cell cycle regulators to gain a more comprehensive understanding of the balance between CMBN and CM proliferation. In conclusion, the loss of *Cdk1* is a potential key regulator of heart regeneration post-infarction. Targeting Cdk1-related pathways could hold therapeutic potential for improving heart regeneration after an MI to reduce HF risks and improve quality of life.

## 5. Materials and Methods

### 5.1. Generation of Cardiac-Specific Cdk1-Knockout Mice

All animal usage was performed in accordance with the institutional animal care guidelines of University Health Network (AUP 1379; Canadian Council in Animal Care). As outlined in the ensemble website (http://useast.ensembl.org/index.html), the murine *Cdk1*-202 mRNA (strain reference CL57BL6) is located on chromosome 10 on the reverse strand. This transcript has 9 exons, of which 7 are coding exons. The length of the *Cdk1*-202 transcript is 1253 bp that codes for 297 residues and has a molecular weight of 34.1 kDa. To examine the potential role of *Cdk1* in mice, a constitutive cardiac-specific *Cdk1* knockout (*Cdk1KOc*) was generated. This was accomplished by crossing *Cdk1^f/f^* mice (129S(B6N)-*Cdk1* ^tm1Eddy^/J; Jackson laboratory, Bar Harbor, ME, USA) [46] with transgenic mice carrying the a-MHC Cre recombinase construct (Jackson laboratory, B6.FVB-Tg(*Myh6*-cre)2182Mds/J) [47]. During generation of the *Cdk1^f/f^* strain, mice were crossed to mice expressing FLP1 recombinase to excise the neomycin selection cassette. The cardiac-muscle-specific α-myosin heavy chain 6 promoter drives the expression of Cre recombinase at birth and results in a recombination efficiency of more than 90% [47]. Cre recombinase fuses together the flanking *loxP* site of the engineered *Cdk1^f/f^* gene at either side of exon 3, thereby deleting the translational initiation site. *Cdk1KOc* had exon 3 from *Cdk1* deleted in CM from day E14, which leads to a reading frameshift mutation that induces a premature translational stop of the *Cdk1* mRNA. These mice did not express functional forms of *Cdk1*.

### 5.2. Isolation of DNA and Genotyping

DNA isolated from fresh tail snips (<5 mm) was used for genotyping. Samples were incubated in 300 μL of 50 mM NaOH for 2 h at 80 °C while rocking, and neutralized with 25 μL 1.0 M HCl in 700 μL H_2_O. Samples were vigorously vortexed, centrifuged for 13,000 rpm at 10 min, and stored at 4 °C. A total of 0.5 μL of DNA sample was used per PCR reaction with the following primers: *Cdk1* forward 5′-CCAGGGTGA CCTTGTGCT-3′; Cdk1 reverse 5′-AGCCTGCCTCCACTTCCA-3′; *Cdk1* Post-Cre forward 5′-GCACTCGGCCTCTAAGCTC-3′; *Cdk1* Post-Cre reverse 5′-TCCACTTGGGAAAGGTGTTC-3′; Myh6-Cre forward 5′-ATGACAGACAGATCCCTCCT ATCTCC-3′; Myh6-Cre reverse 5′-CTCATCA CTCGTGCATCATCGAC-3′. PCR analysis was carried out with the Applied Biosystems ProFlex PCR system by Life Technologies (4484073; Thermo Fisher Scientific, Mississauga, ON, CA); Cdk1 wild-type allele, 299 bp; Cdk1 floxed allele, 320 bp; Cdk1 Post-Cre, 838 bp (Figure 3B). Myh6-Cre transgene, 300 bp; and Myh6-Cre internal positive control, 200 bp.

### 5.3. Coronary Artery Ligation

To create an MI, permanent ligation of the left descending coronary artery was performed in in 10–12-week-old male and female *Cdk1KOc* and *control* mice. At the time of surgery and at 24 h post-MI, a single subcutaneous dose of buprenorphine (30 mg/g body weight) (B9275; Sigma-Aldrich, Oakville, ON, CA) was given as described previously [42].

### 5.4. Echocardiography

Echocardiography was performed in anesthetized mice pre-MI and at 21 d post-MI.

### 5.5. DNA Synthesis, TUNEL, ROS Assays, and Three-Dimensional Immunofluorescence Microscopy

For EDU-labeling experiments, EDU (160 mg/kg body weight cumulative dosage) was intraperitoneally injected twice at 4 d post-MI. Hearts were sampled 2 h after the second injection. For detection of incorporated EDU, the Click-iT™ EdU Cell Proliferation Kit for Imaging (Alexa Fluor™ 488 dye; C10337; Thermo Fisher) was employed according to the manufacturer’s specifications.

Detection of fragmented genomic DNA (apoptosis assay) was performed by terminal deoxynucleotidyl transferase-mediated dUTP nick-end labeling according to the manufacturer’s instructions (no. 1684795910; Roche, Mississauga, ON, CA) [53]. For immunofluorescence measurements of cell cycle distribution, and CMBN, 3 independent fields of approximately of 0.25 mm^2^ per left ventricular (LV) sample were counted on 3 consecutive sections. Then, 3D-reconstruction of digital microphotographs were performed using Imaris Imaging software, version 10.0.

### 5.6. Primary Mouse Neonatal Ventricular Cardiomyocyte Isolation

Hearts from 1-day-old neonatal *Cdk1KOc* mice and controls were isolated as described [65].

### 5.7. Preparation of Protein Extracts from Left Ventricular Heart Tissue Samples

Only left ventricular (LV) specimens were used from neonatal hearts derived from *Cdk1KOc* mice and controls, and they were snap frozen and stored at −80 °C. Preparation of total protein extracts was performed as described previously [42].

### 5.8. Western Blotting

Western blotting was carried out as described previously [42]. The antibodies employed in this study are summarized in the Supplementary Table S1.

### 5.9. Total RNA Isolation, Reverse Transcription, and Quantitative Real-Time PCR Assays

Total RNA isolation, reverse transcription, and quantitative real-time PCR assays were performed as described previously [42]. The following oligonucleotide primers were used:

α-smooth muscle actin (*Acta2*) forward 5′-CTGACAGAGGCACCACTGAA-3′, reverse 5′-CAT CTCCAGAGTCCAGCACA-3′. β-Actin forward 5′-GGCTGTATTCCCCTCCATCG-3′, reverse 5′-CCAGTTGGTAACAATGCCATGT-3′. ANP forward 5′-GCTTCCAGGCCATATTGGAG-3′, reverse 5′-GGGGGCATGACCTCATCTT-3. BNP forward 5′- GAGGTCACTCCTATCC TCTGG-3′, reverse 5′-GCCATTTCCTCCGACTTTTCTC-3′. α-MHC forward 5′-GCCCAGTA CCTCCGAAAGTC-3′, reverse 5′-GCCTTAACATACTCCTCCTT GTC -3′. β-MHC forward 5′-ACTGTCAACACTAAG AGGGTCA-3′, reverse 5′-TTGGATGATTTGATCTTCCAGGG-3′.

### 5.10. Cell Cycle RT-qPCR Array

RNA was purified from the hearts of neonatal wild-type mice as described previously [28]. Equal amounts of RNA (400 ng/sample) were converted into cDNA using the First Strand cDNA Synthesis kit (Qiagen, Toronto, ON, CA). The Cell Cycle PCR Array was obtained from Qiagen (330171) and used to profile the expression of 87 cell cycle genes, 5 housekeeping genes, controls for genomic DNA contamination, and the efficiency of both the reverse transcriptase and PCR reaction as described previously [42].

### 5.11. Gene Expression Analysis and Bioinformatics

Affymetrix Mouse Gene 2.0 ST expression arrays were processed at the Centre for Applied Genomics (Toronto, ON, Canada). Processing of probe level data and subsequent data analyses were performed using GeneSpring (Version 13.2; Agilent Technologies Inc., Santa Clara, CA, USA) as described previously [53]. Genome-wide data from the gene expression microarrays were normalized, filtered on expression (in the range of −5.2 to 3.5), filtered on Error-CV < 50.0 percent, and filtered for genes with significant differences in expression levels (log_2_ fold change ±1.3; *p* < 0.05), followed by determination of statistical significance according to a *t* test with Benjamini–Hochberg correction (log_2_ fold change ±1.3; *p* < 0.01) in wild-type neonatal mice (0 d through 15 d) in comparison to 0 d employing GeneSpring; *n* = 6 to 20 per time point. GSEA and GO term analysis of differentially expressed genes were carried out with GeneSpring. Microarray data were submitted to the ArrayExpress database (http://www.ebi.ac.uk/arrayexpress. Access date 11 January 2019). Accession number: E-MTAB-8168.

RNA-seq samples were processed at the Princess Margaret Genomics Centre, 101 College St., 9-601, Toronto, ON, M5G 1L7, Canada. Briefly, whole-exome RNA-seq was performed using paired-end 150 nucleotide sequencing with 40 million reads. Processing of probe-level data and subsequent data analyses were performed using Strand-NGS software. Genome-wide data were normalized and filtered for genes with significant differences in expression levels (log_2_ fold change ±2.0; *p* < 0.01), followed by determination of statistical significance according to a *t* test with Benjamini–Hochberg correction (log_2_ fold change ±2.0; *p* < 0.01) employing Strand-NGS. The 3D-PCA analysis, Volcano plot, Heatmaps, GSEA, GO, and Pathway analysis of differentially expressed genes were carried out with Strand-NGS. RNA-seq data were submitted to the ArrayExpress database (http://www.ebi.ac.uk/arrayexpress, accessed on 24 September 2024). Accession numbers: E-MTAB-14505 for samples collected 4 days post-MI and E-MTAB-14508 for samples collected 3 weeks post-MI, respectively.

### 5.12. Isolation of Mitochondria and Detection of Oxidative Damage, Antioxidants, and ATP Levels

Isolation of mitochondria and the detection of oxidative damage, antioxidants and ATP levels was carried out as described previously [65].

### 5.13. Statistical Analyses

Statistical analyses were carried out using GraphPad InStat (version 3.1) and GraphPad Prism (GraphPad Software, version 10.3.1; La Jolla, CA, USA). Data are reported as means ± s.e.m. Two-tailed *p*-values of < 0.05 were considered as significant. When groups passed the normality test, we performed data evaluation between 2 groups by an unpaired Student’s t-test. The statistical significance of 4 groups was calculated using a one-way analysis of variance (ANOVA) and Tukey–Kramer multiple comparison post-test.

We calculated experimental power with GraphPad Statmate (GraphPad Software, version 1, La Jolla, CA, USA). For example, for the FS data presented in Figure 6L, we used the value of 1 for the standard deviation (S.D.) for *Cdk1KOc* mice versus controls post-MI, together with a significance level alpha = 0.01 (two-tailed) and 80% power. Therefore, at least *n* = 4 per group was required in each experiment and that was increased to *n* = 6 per group.

Survival analysis was performed using a conservative log-rank test (Mantel–Cox). For calculation of experimental power, the expected proportion of being event-free or surviving was 20% for the *Pkm2KOi* mice versus controls. Thus, a sample size of *n* = 20 for *Cdk1KOc* mice and 24 for the controls post-MI in each group provided 80% power to detect an increase in survival proportion of 0.115 with a significance level alpha of 0.01 (two-tailed). For the proliferation data (Figure 5), we used an S.D. value of 3.5 and *n* = 6 per group in each experiment for *Cdk1KOc* mice versus the controls post-MI. This resulted in an 80% power to detect a difference between means of 5.78 with a significance level (alpha) of 0.01 (two-tailed).

## Data Availability

All data and reagents in this publication can be shared upon request to the Phyllis Billia.

## Supplementary Materials

The following supporting information can be downloaded at: https://www.mdpi.com/article/10.3390/ijms251910824/s1.
