# Supplementary material for: Cdk1 Deficiency Extends the Postnatal Window of Cardiomyocyte Proliferation and Restores Cardiac Function after Myocardial Infarction
Source: Int J Mol Sci. 2024 Oct 9;25(19):10824. doi: 10.3390/ijms251910824 (PMC13231826; doi:10.3390/ijms251910824)
Supplement: Supplementary file 1 [file ijms-25-10824-s001.zip › ijms-3205649-supplementary.pdf]

Supplementary Table S1: Antibodies employed for IF and WB.

Gene name	Catalog No.	Vendor	Application	Dilution Factor
**α-actinin sarcomeric**	A7811	Sigma-Aldrich	IF	50
**ATP5a**	ab14748	Abcam	WB	1000
**AurkB**	A5102	Sigma-Aldrich	IF	50
**Cat**	14097	Cell Signaling	WB	1000
**Cdk1**	28439	Cell Signaling	WB	1000
**Cdk2**	2546	Cell Signaling	WB	1000
**Cdk4**	2906	Cell Signaling	WB	1000
**Cyclin A2**	4656	Cell Signaling	WB	1000
**Cyclin B1**	4138	Cell Signaling	WB	1000
**Cyclin D1**	2978	Cell Signaling	WB	1000
**Cyclin D2**	3741	Cell Signaling	WB	1000
**Cyclin D3**	2936	Cell Signaling	WB	1000
**Cyclin E**	4129	Cell Signaling	WB	1000
**DJ-1**	N/A	custom-made	WB	1000
**Drp1**	5391	Cell Signaling	WB	500
**E2f1**	sc-251	Santa Cruz	WB	500
**E2f4**	sc-866	Santa Cruz	WB	500
**Erk1/2**	4695	Cell Signaling	WB	1000
**Erk1/2.Pi-T202/T204**	4370	Cell Signaling	WB	1000
**H3.Pi-S28**	H9908	Sigma-Aldrich	IF	50
**Hk2**	2867	Cell Signaling	WB	1000
**Mdm2**	M8558	Sigma-Aldrich	WB	500
**Mef2a**	sc-33672	Santa Cruz	WB	500
**Mfn2**	8482	Cell Signaling	WB	1000
**Mlc2v**	3672	Cell Signaling	WB	1000
**Mlc2v.Pi-S19**	3671	Cell Signaling	WB	1000
**Npm1**	B0556	Sigma-Aldrich	WB	1000
**Nqo1**	ab34173	Abcam	WB	1000
**Opa1**	ab42364	Abcam	WB	1000
**p19** ***Cdkn2d***	sc-1665	Santa Cruz	WB	1000
**p21** ***Cdkn1a***	556431	BD Biosciences	WB	1000
**p27** ***Cdkn1b***	610241	BD Biosciences	WB	1000

**p38**	8690	Cell Signaling	WB	1000
**p38.Pi-T180/T182**	4511	Cell Signaling	WB	1000
**p53**	BML-SA293-0050	Enzo	WB	1000
**p130**	610261	BD Biosciences	WB	1000
**Pcm1**	sc-67204	Santa Cruz	IF	50
**Pink1**	3929-100	BioVision	WB	1000
**Rb**	554136	BD Biosciences	WB	1000
**Ryr2**	PA5-38329	Thermo/Fisher Scientific	WB	1000
**Sdh1**	Ab151684	Abcam	WB	1000
**Serca2a**	9580	Cell Signaling	WB	1000
**Sod2**	13194	Cell Signaling	WB	1000
**Tubulin**	2146	Cell Signaling	WB	1000

Supplementary Figure S1

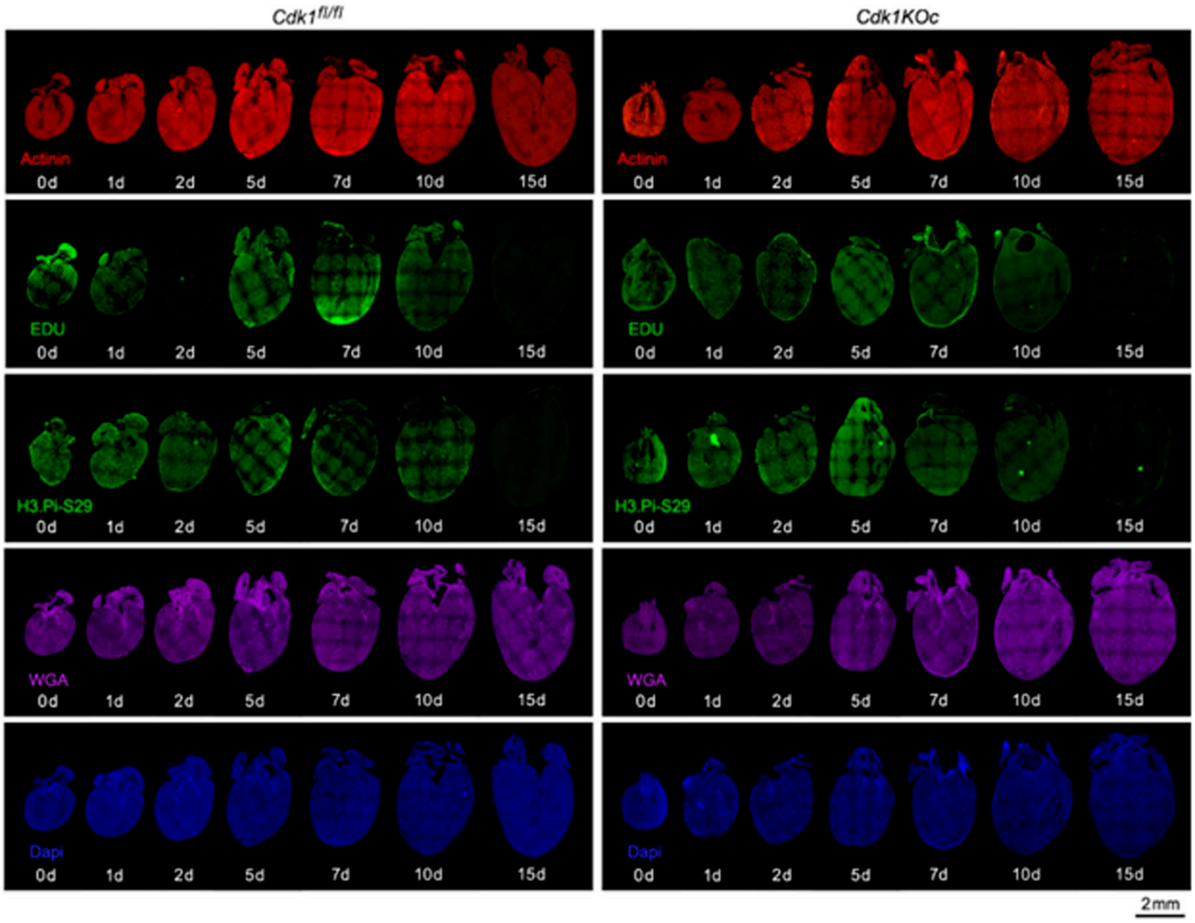

Figure S1. Histological representation of whole hearts from *CdklKOc* and *controls* during the first 15d after birth. Fixed tissue sections were labelled with the nuclear S-phase marker EDU (green) in conjunction with antibodies to CM-specific cytoplasmic marker α-actinin (red), the nuclear M-phase marker H3.Pi-S28 (green), WGA to detect the ECM (purple), and DAPI to stain genomic DNA (blue).

Supplementary Figure S2

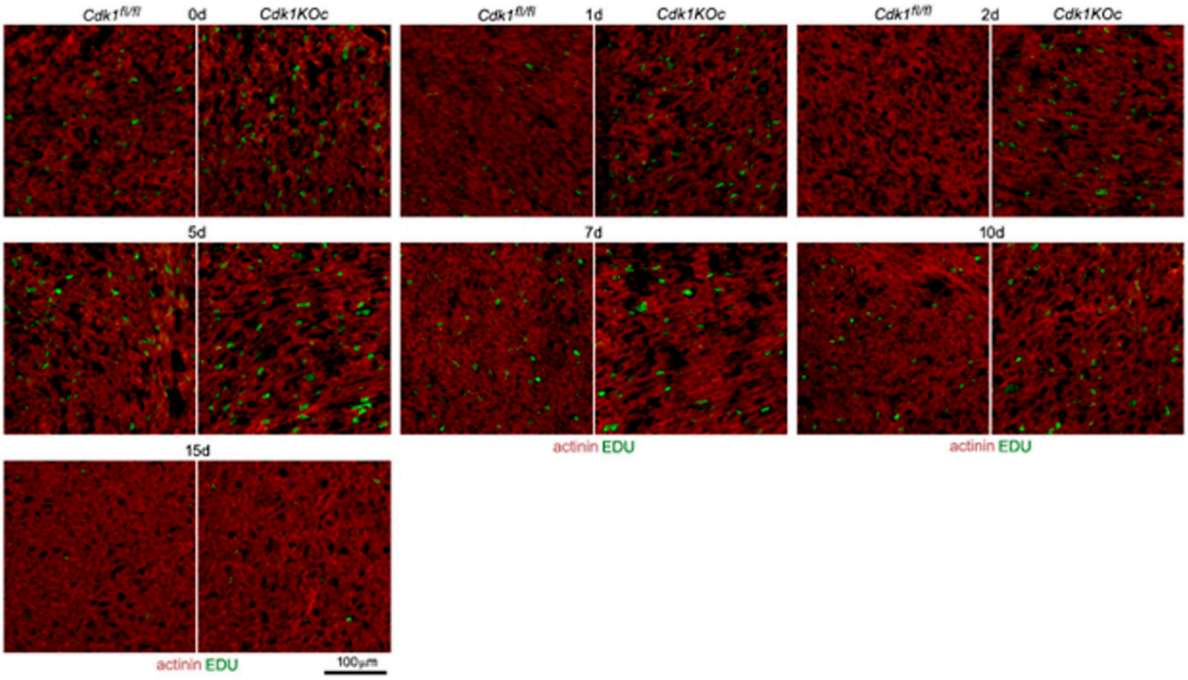

Figure S2. Histological sections of hearts derived from *Cdk1KOc* and *controls* depicting DNA replication during early post-natal period. Fixed tissue sections were labelled with EDU (green) in conjunction with antibodies to α-actinin (red).

Supplementary Figure S3

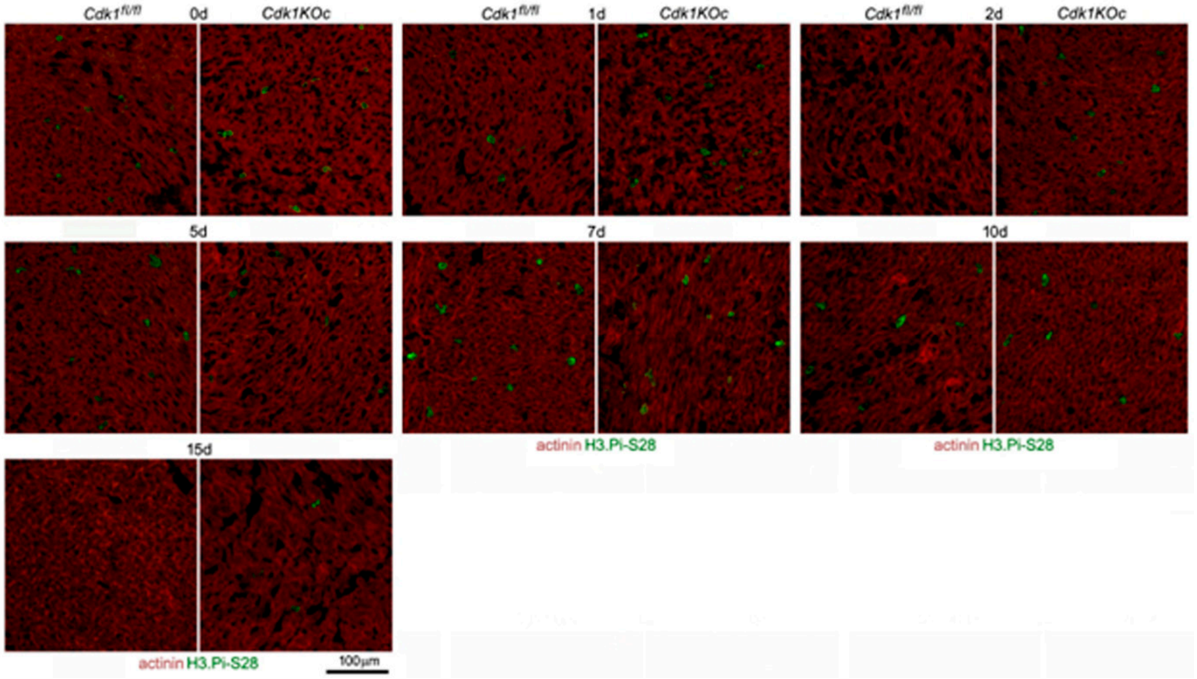

Figure S3. Histological heart sections from *Cdk1KOc* and *controls* showing mitotic figures during the early postnatal period. Specimen were stained with antibodies to nuclear H3.Pi-S28 (green) and α-actinin (red).

Supplementary Figure S4

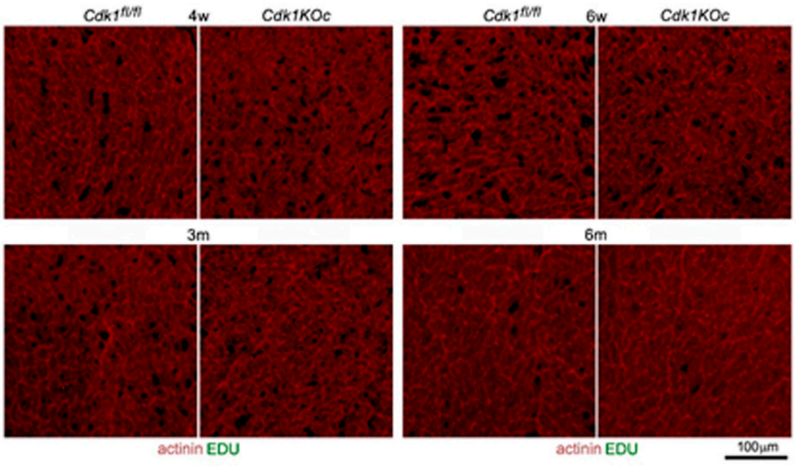

Figure S4. Histological heart sections demonstrate absence of DNA replication in young adult mice at 4w and 6w of age. Specimen derived from *Cdk1KOc* and *controls* were co-stained with EDU (green) and with antibodies to α-actinin (red).

Supplementary Figure S5

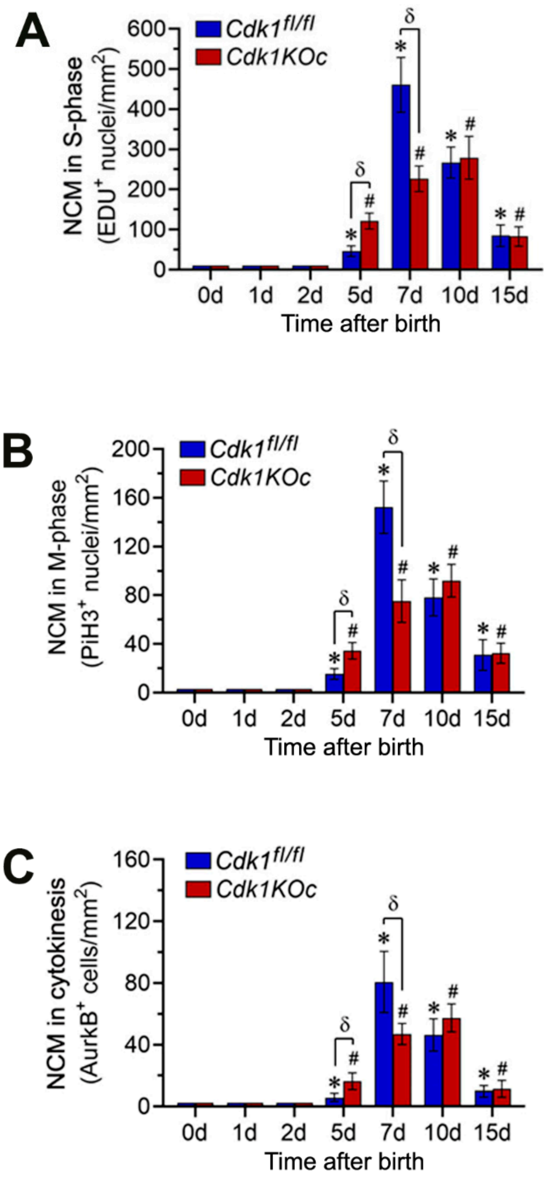

Figure S5. Cdk1 ablation increases non-cardiomyocyte (NCM) proliferative at postnatal day 5 in vivo.
(A) Analysis of NCM in S-phase in response to Cdk1 ablation. Heart samples were collected from *Cdk1KOc* and *controls* at the indicated timepoints. NCMs were analyzed by confocal immunofluorescence microscopic employing the S-phase marker EDU (green).
(B) Analysis of NCM in M-phase in *Cdk1KOc* mice and *controls.* Fixed tissue sections were stained with antibodies to the nuclear M-phase marker H3.Pi-S28 (green),
(C) Analysis of NCM in cytokinesis in the absence of Cdk1. Fixed tissue sections were immuno-stained for with antibodies to aurora kinase B (AurkB), a mid-body-specific cytokinesis marker. Data are mean ± S.D. $n=4$ biological replicates. *$P<0.01$ vs. controls/0d. #$P<0.01$ vs. *Cdk1KOc* at 0d. δ$P<0.001$. φ$P<0.01$.

Supplementary Figure S6

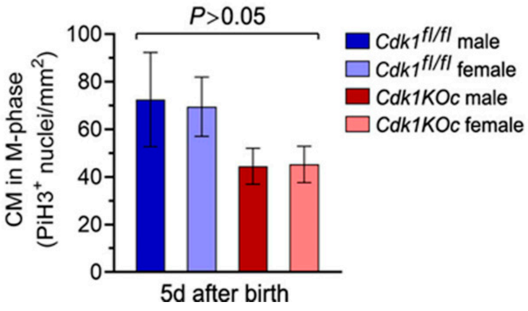

Figure S6. Cell-cycle activation in CM from *Cdk1KOc* mice and *controls* is sex-independent. Quantification of male and female CM in M-phase in *Cdk1KOc* and *controls* was conducted by confocal immunofluorescence microscopy. Data are mean ±s.e.m.

Supplementary Figure S7

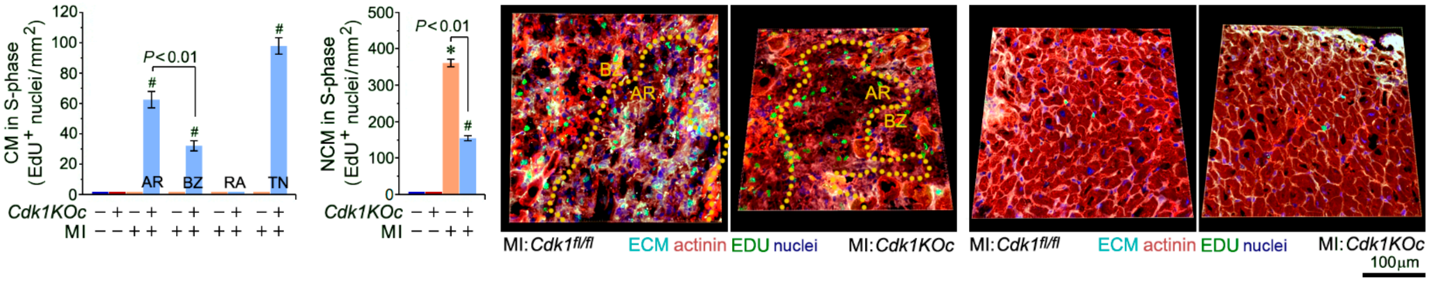

Supplementary Figure S8

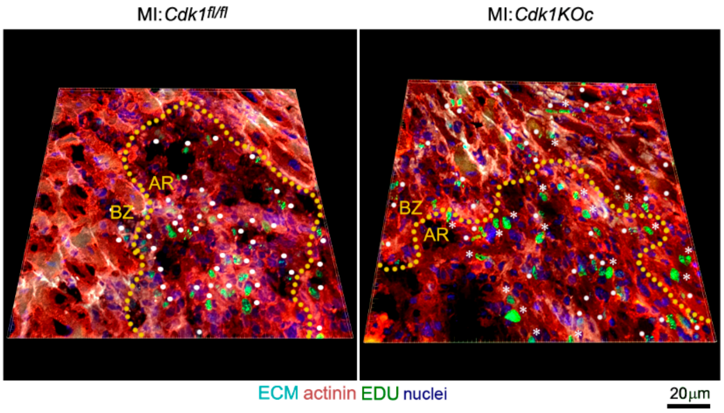

Supplementary Figure S9

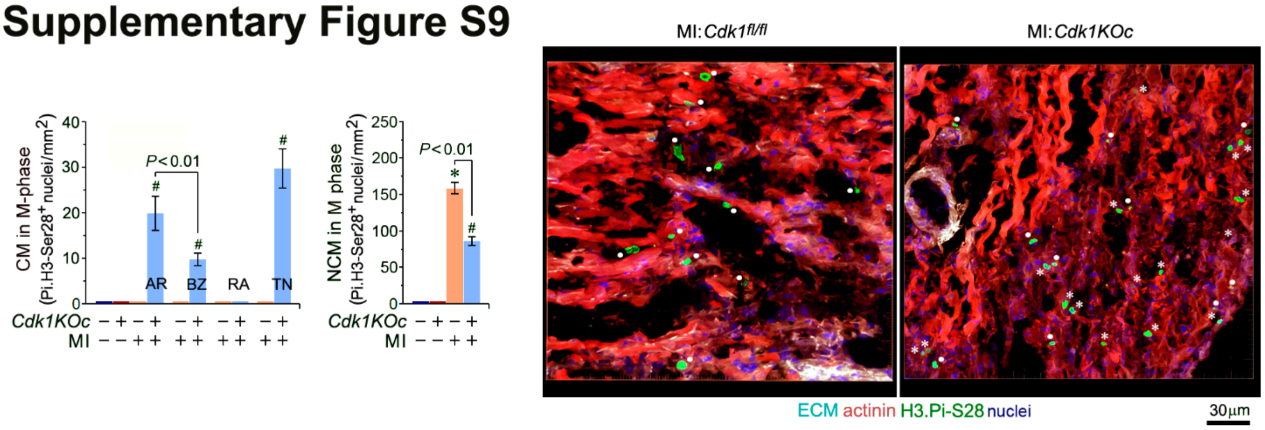

Supplementary Figure S10

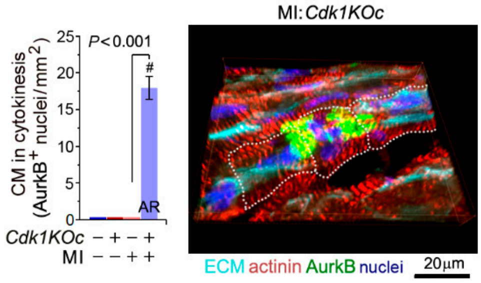

Figure 2G

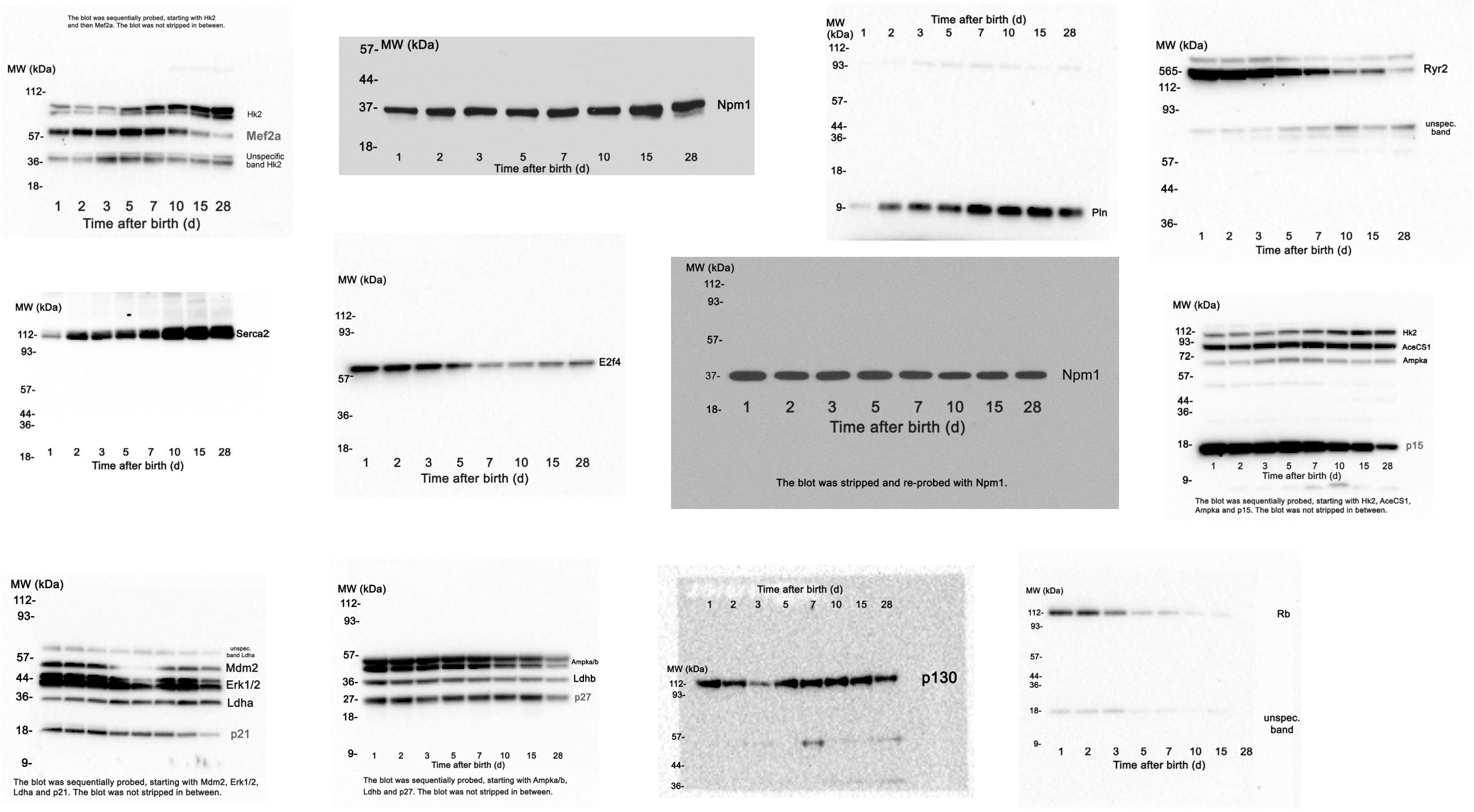
The blot was sequentially probed, starting with Hk2 and then Mef2a. The blot was not stripped in between.
MW (kDa)
Hk2
Mef2a
Unspecific band Hk2
Time after birth (d)
Npm1
Pln
Ryr2
unspec. band
Serca2
E2f4
The blot was stripped and re-probed with Npm1.
Hk2
AceCS1
Ampka
p15
The blot was sequentially probed, starting with Hk2, AceCS1, Ampka and p15. The blot was not stripped in between.
unspec. band Ldha
Mdm2
Erk1/2
Ldha
p21
The blot was sequentially probed, starting with Mdm2, Erk1/2, Ldha and p21. The blot was not stripped in between.
Ampka/b
Ldhb
p27
The blot was sequentially probed, starting with Ampka/b, Ldhb and p27. The blot was not stripped in between.
p130
Rb
unspec. band

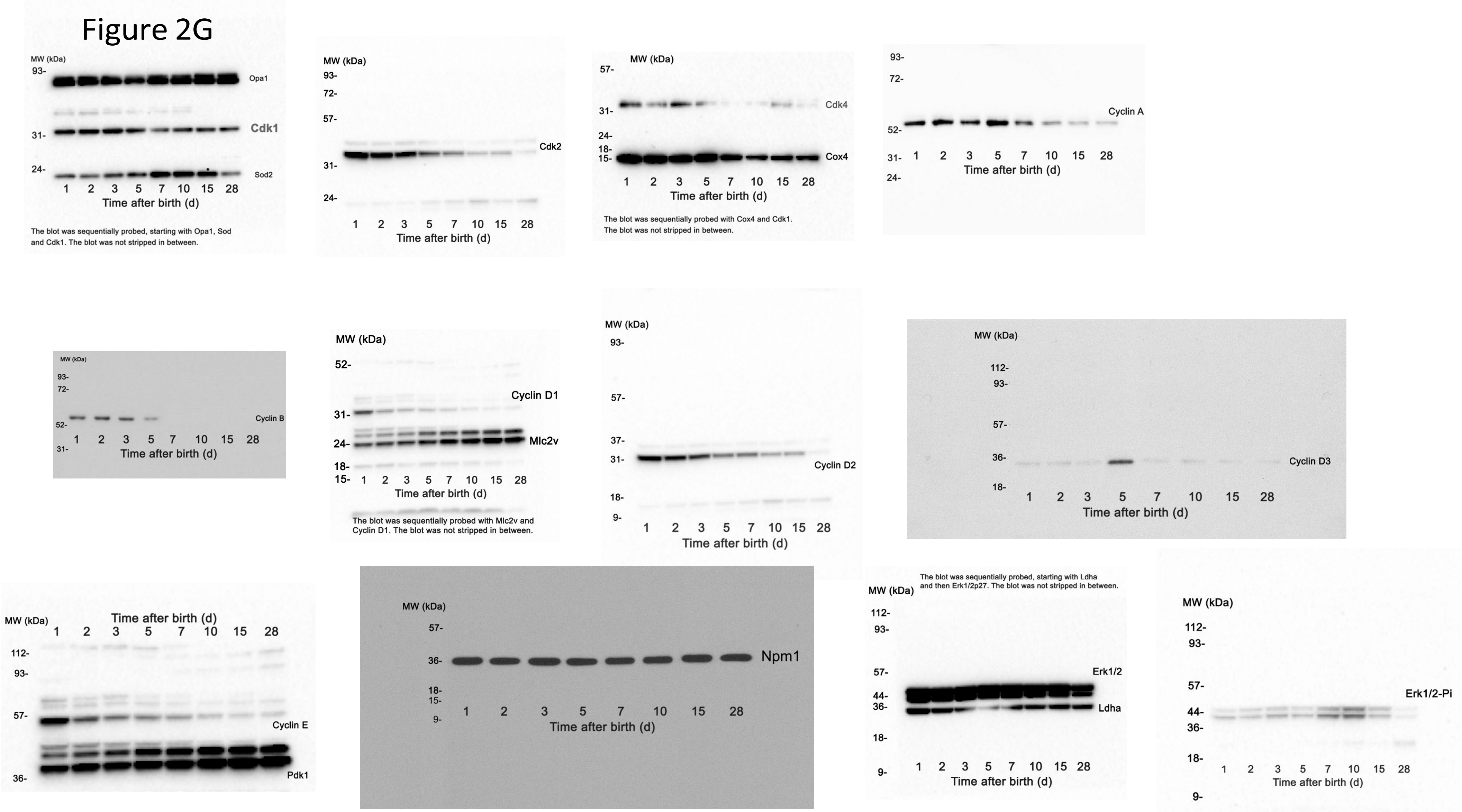
Figure 2G
MW (kDa)
Opa1
Cdk1
Sod2
Time after birth (d)
The blot was sequentially probed, starting with Opa1, Sod and Cdk1. The blot was not stripped in between.
Cdk2
Cdk4
Cox4
The blot was sequentially probed with Cox4 and Cdk1. The blot was not stripped in between.
Cyclin A
Cyclin B
Cyclin D1
Mlc2v
The blot was sequentially probed with Mlc2v and Cyclin D1. The blot was not stripped in between.
Cyclin D2
Cyclin D3
Cyclin E
Pdk1
Npm1
The blot was sequentially probed, starting with Ldha and then Erk1/2p27. The blot was not stripped in between.
Erk1/2
Ldha
Erk1/2-Pi
1 2 3 5 7 10 15 28

Figure 2G

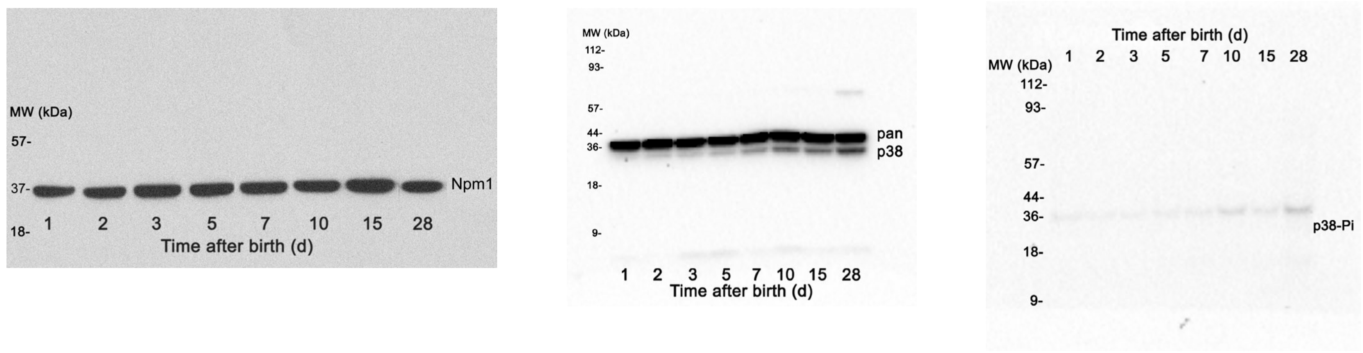

MW (kDa)
57-
37-
18-
Npm1
1 2 3 5 7 10 15 28
Time after birth (d)
MW (kDa)
112-
93-
57-
44-
36-
18-
9-
pan
p38
1 2 3 5 7 10 15 28
Time after birth (d)
Time after birth (d)
MW (kDa)
1 2 3 5 7 10 15 28
112-
93-
57-
44-
36-
18-
9-
p38-Pi

Figure 3C

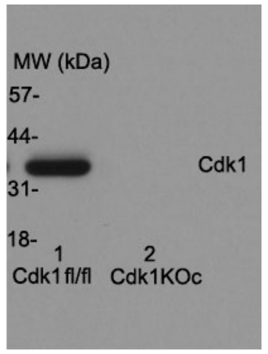
MW (kDa)
57-
44-
Cdk1
31-
18-
1
2
Cdk1 fl/fl
Cdk1KOc

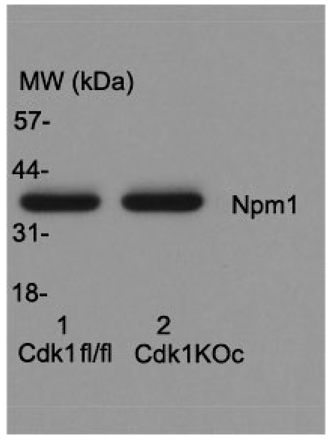
MW (kDa)
57-
44-
Npm1
31-
18-
1
2
Cdk1 fl/fl
Cdk1KOc

Figure 5O

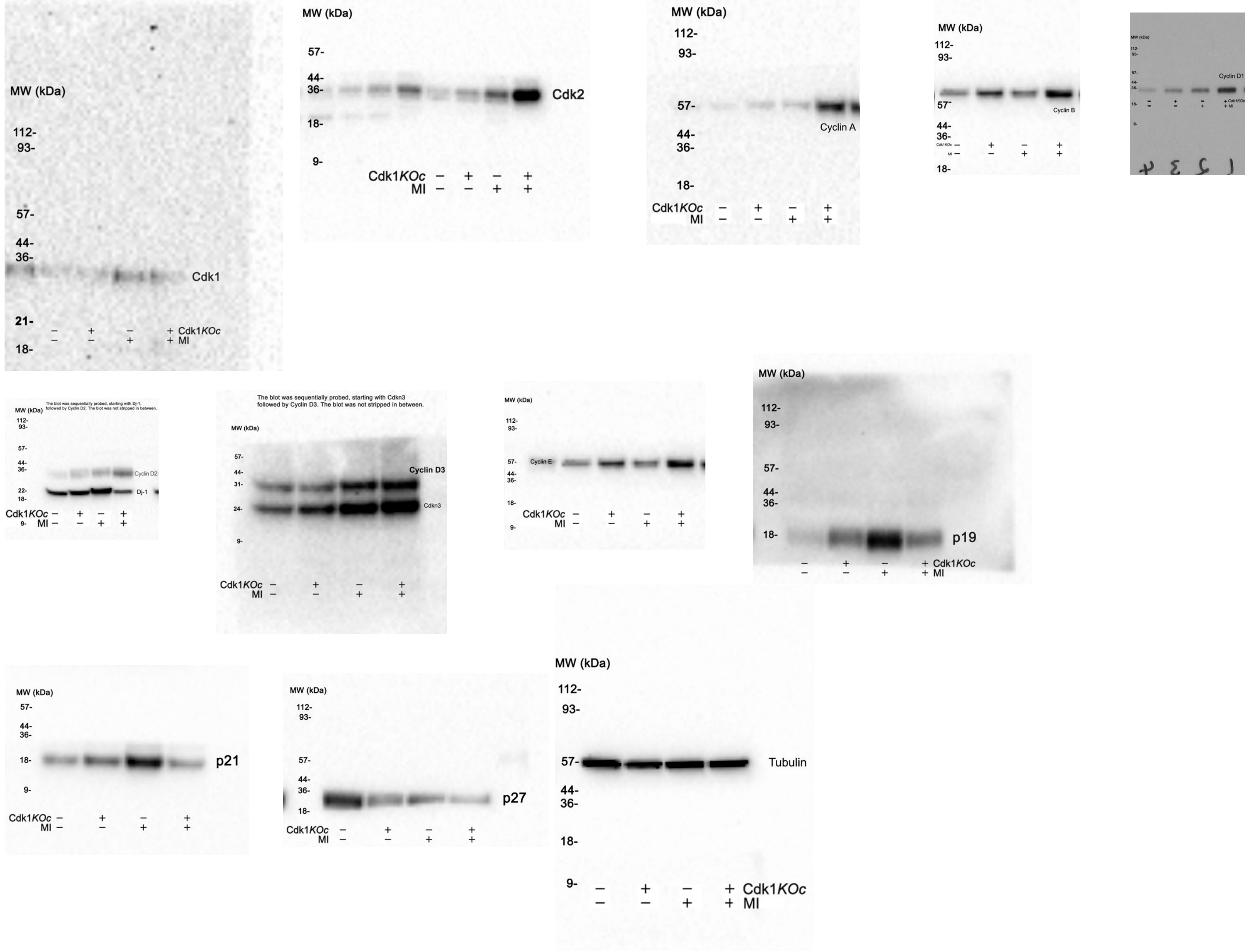
MW (kDa)
Cdk1
Cdk1KOc
MI
Cdk2
Cyclin A
Cyclin B
Cyclin D1
The blot was sequentially probed, starting with Dj-1, followed by Cyclin D2. The blot was not stripped in between.
Cyclin D2
Dj-1
The blot was sequentially probed, starting with Cdkn3 followed by Cyclin D3. The blot was not stripped in between.
Cyclin D3
Cdkn3
Cyclin E
p19
p21
p27
Tubulin

Figure 5O

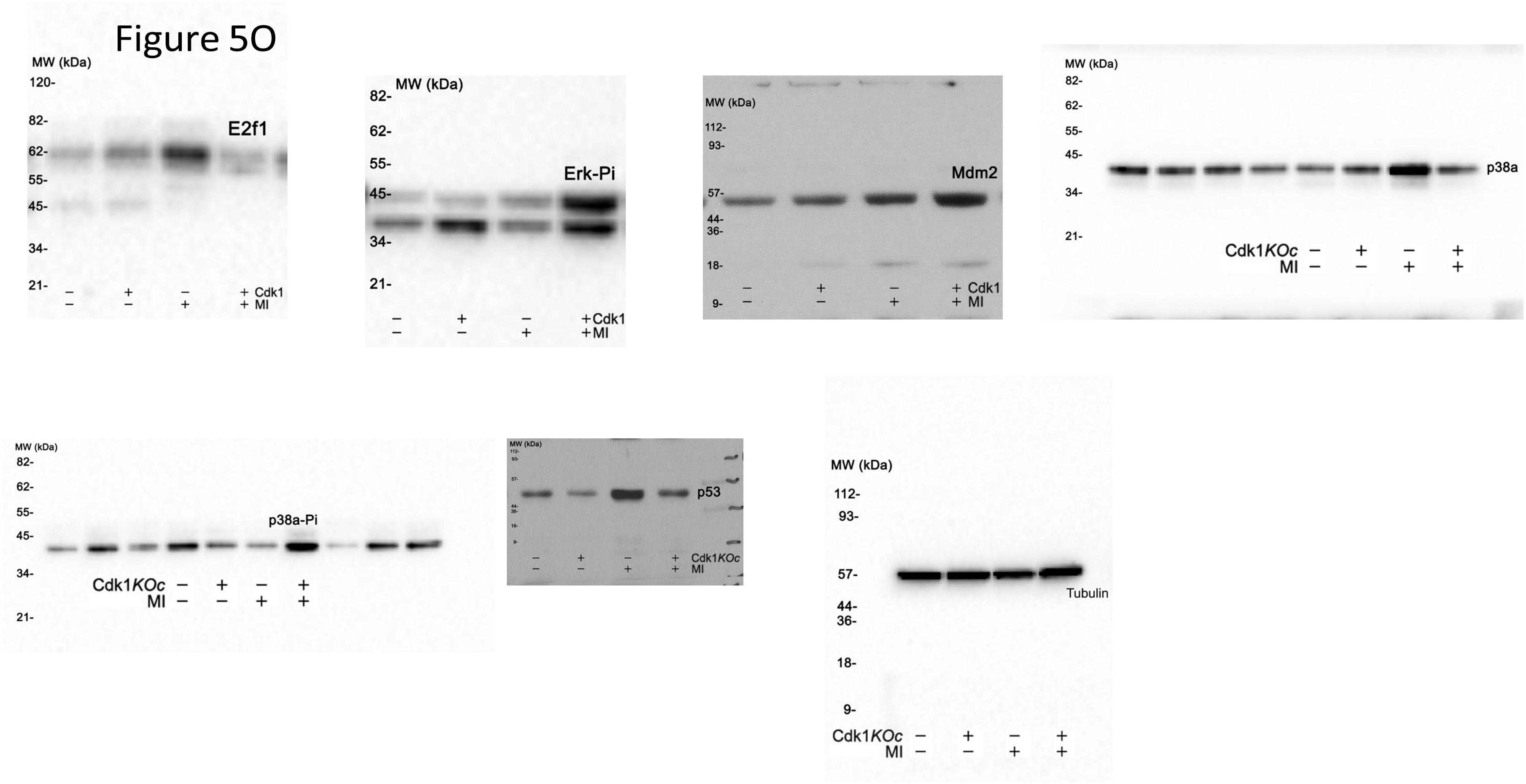
MW (kDa)
E2f1
Cdk1
MI
Erk-Pi
Mdm2
p38a
Cdk1KOc
p38a-Pi
p53
Tubulin
120-
112-
93-
82-
62-
57-
55-
45-
44-
36-
34-
21-
18-
9-

Figure 5S

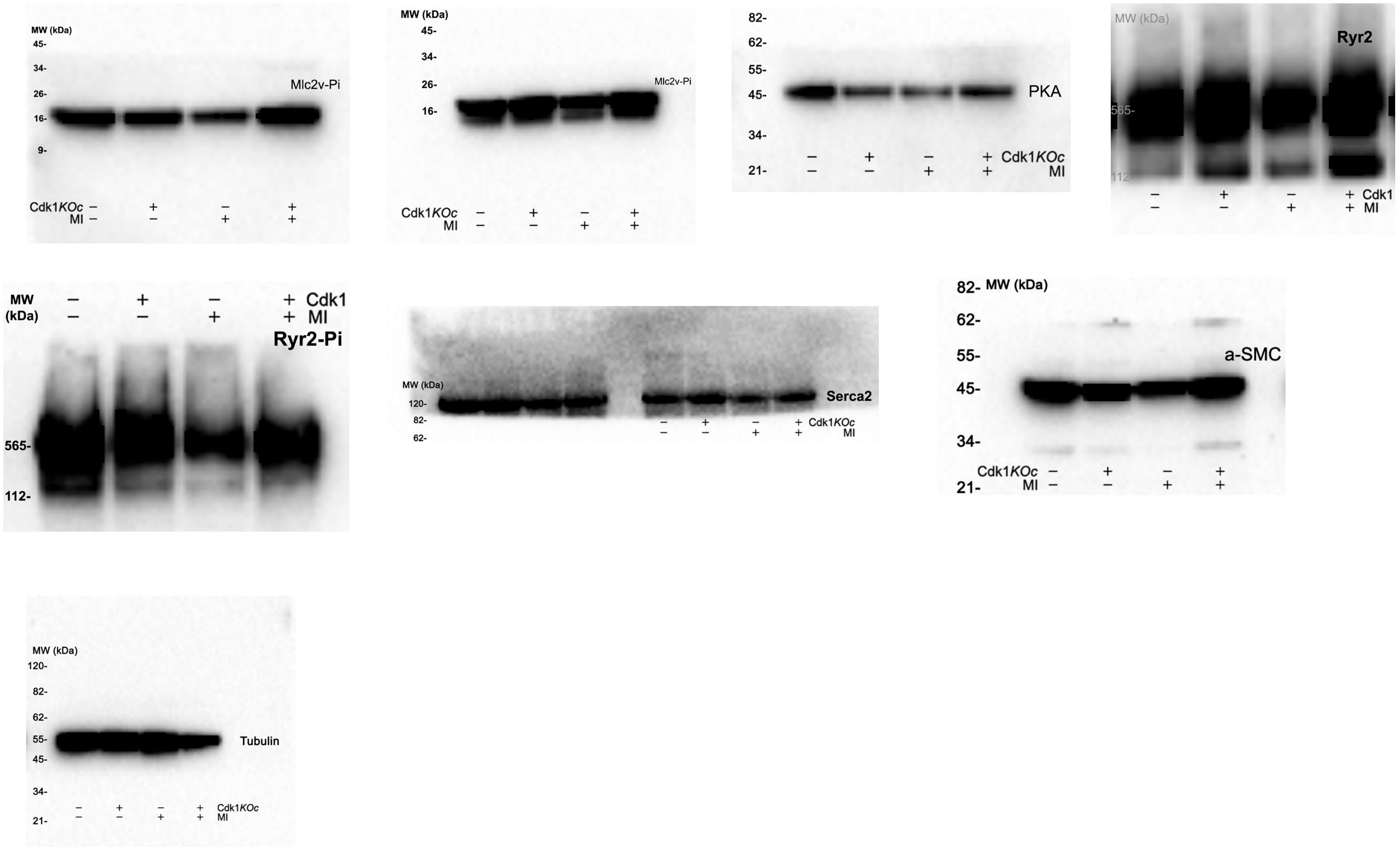
MW (kDa)
Mlc2v-Pi
Cdk1*KOc* − + − +
MI − − + +
Mlc2v-Pi
PKA
Cdk1*KOc*
MI
Ryr2
Cdk1
MI
Ryr2-Pi
Serca2
a-SMC
Tubulin

Figure 6B

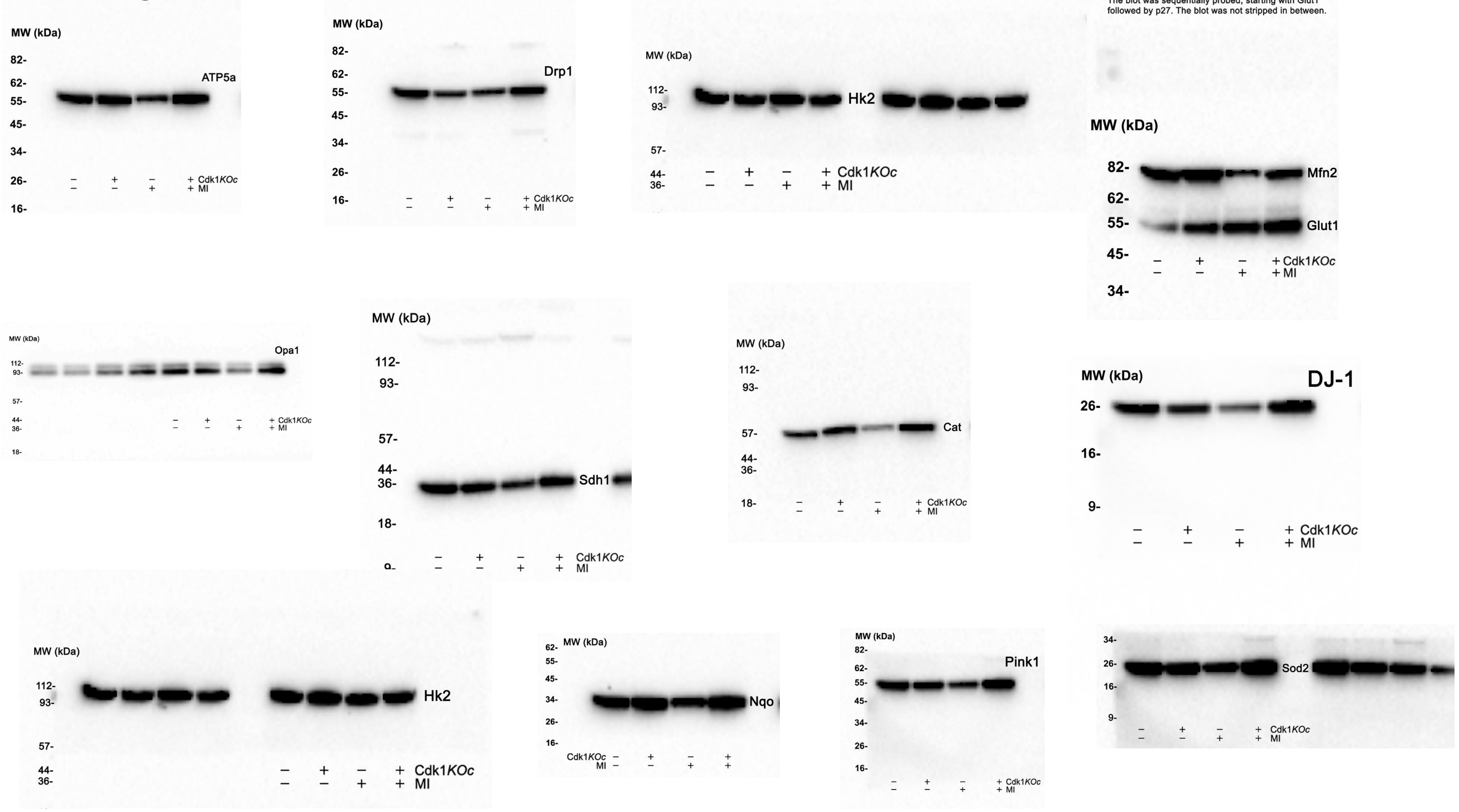

MW (kDa)
82-
62-
55-
45-
34-
26-
16-
ATP5a
Drp1
Hk2
Cdk1KOc
MI
The blot was sequentially probed, starting with Glut1 followed by p27. The blot was not stripped in between.
Mfn2
Glut1
Opa1
112-
93-
57-
44-
36-
18-
9-
Sdh1
Cat
DJ-1
Nqo
Pink1
Sod2
